# Health consequences of spousal health shocks: evidence from a matched event study in the United States

**DOI:** 10.1093/aje/kwag178

**Published:** 2026-07-24

**Authors:** Ah-Reum Lee, Ashwin Kotwal, Jacqueline M Torres

**Affiliations:** Department of Epidemiology and Biostatistics, University of California San Francisco, San Francisco, CA, United States; Division of Geriatrics, University of California San Francisco, San Francisco, CA, United States; Department of Epidemiology and Biostatistics, University of California San Francisco, San Francisco, CA, United States

**Keywords:** spouses, acute disease, outcome assessment, morbidity, mental health

## Abstract

Major disease diagnoses can create shocks that ripple through households. Population-level evidence in the US on how spouses and partners fare after a partner’s major diagnosis remains sparse and is often limited to caregiver or employment-related outcomes. Using the US Health and Retirement Study (1992-2020), we evaluated population-level health spillovers to spouses after a partner’s first diagnosis of cancer, stroke, or heart disease, employing a stacked event-study design with matching (*n* = 29 166). Outcomes included subjective health, doctor-diagnosed conditions, limitations in basic and instrumental activities of daily living (ADL/IADL), and health behaviors and use of preventive care. We evaluated heterogeneity by sociodemographic and economic conditions. Spouses of newly diagnosed patients experienced modest but persistent declines: more frequent reports of poor health ($\boldsymbol{\beta}$ = .02, 95% CI, 0.00, 0.03), more doctor-diagnosed conditions ($\boldsymbol{\beta}$ = .05, 95% CI, 0.01, 0.08), and higher depressive symptoms ($\boldsymbol{\beta}$ = .08, 95% CI, 0.01, 0.16). Effects were greater among women whose male partners received a diagnosis and among spouses with lower education or fewer assets. Health and preventive care behaviors were largely unchanged. Findings motivate early, spouse-focused support and underscore the need for targeted interventions for spouses at highest risk.

## Introduction

Health shocks—sudden serious diagnoses or acute events—are common and disruptive to the entire family. In the United States alone, over 2 million new cancer cases are projected in 2025, and more than 795 000 people experience a stroke each year.^1^^,^^2^ Spousal health shocks have been linked to higher partner mortality risk,^3-5^ and elevated psychiatric and suicide risk after cancer, especially in the first year.^6-9^

Spousal spillovers may arise through linked lives,^10^ whereby a partner’s serious illness alters shared routines, emotional states, caregiving roles, time and financial constraints,^10-15^ and spouses’ use of preventive or routine care due to increased time devoted to care coordination and daily assistance.^16-21^ Stress and worry may also worsen sleep and mental health and contribute to physiologic wear and tear, including allostatic load, through disruption of the hypothalamic-pituitary-adrenal axis and immune responses, thereby increasing cardiometabolic risk.^22-25^

Thus far, much of the evidence on spillovers of major health shocks on spousal partners comes from non-US, primarily European, settings. Most US studies of within-couple spillovers have focused on employment outcomes such as labor supply, earnings, and financial strain after a partner’s illness, disability, or hospitalization.^26^^,^^27^ Fewer studies assess spouses’ health outcomes, and these typically restrict samples to spouses who self-identify as caregivers, documenting elevated depression,^3^^,^^28^^‑^^34^ sleep disturbance,^35^ cardiometabolic risk,^3^^,^^36^ and cognitive decline.^37^ However, caregiver-based studies may miss broader spousal effects because conditioning on caregiving, a post-shock outcome, excludes spouses affected by the shock who do not report caregiving and may over-represent the most severe cases. Effects may also differ in the US context due to differences in healthcare systems, the social safety net (including paid leave), as well as the landscape of long-term services and supports.

In this study, we estimated the spillover effects on spouses’ health following a partner’s major health shock among older US couples. Health shocks are defined as the new onset of serious, clinically salient conditions common in later life and likely to require substantial adjustment within the household: stroke, cancer, and heart disease. We hypothesized that a partner’s first health shock would lead to an immediate and sustained deterioration in spouses’ health, reflected in higher depressive symptoms and worsening functional limitations. We further hypothesized that these shocks would be associated with short-run reductions in spouses’ preventive care use and adverse changes in health behaviors, including increased alcohol and tobacco use. Finally, we expected these spillover effects to vary by gender and socioeconomic resources. Impacts may be larger and more persistent among women, and among spouses with lower education and fewer assets, who have fewer economic and social buffers against the consequences of a major health shock; however, given evidence that men often derive larger health benefits from marriage, they may also experience negative spillovers when a partner’s illness disrupts these protective pathways.

## Methods

### Study sample

We used the Health and Retirement Study (HRS), a nationally representative, biennial panel of US adults aged 51+ that survey respondents and their spouses or partners regardless of spouse age, following multiple entry cohorts from 1992-2020. Analyzes drew on the RAND HRS Longitudinal File (2020 v1), linked to core HRS files to construct a household–wave panel.

The analytic sample consisted of married or cohabiting respondents observed between 1992 and 2020 (*n* = 42 405). We excluded respondents who were not partnered at first interview (*n* = 12 308); those who later separated, divorced, or were widowed (7.8%) remained in the sample if they continued to contribute the required consecutive waves of data. To focus on incident events, individuals reporting a baseline history of cancer, stroke, or heart disease were excluded (*n* = 6723). We defined cardiac diagnoses using the heart disease measure (including heart attack, coronary heart disease, angina, congestive heart failure, or other heart problems) rather than a more acute diagnosis such as heart attack, because the HRS does not consistently capture lifetime heart attack history at baseline before 2010. Although recent heart attack since the last interview is observed across waves, it cannot distinguish incident events from pre-baseline history. In robustness tests, we estimated models separately for cancer, stroke, and heart disease to assess whether spillovers differed by diagnosis. We also attempted to estimate the model using more acute cardiac event definitions (eg, heart attack), but our stacked event-study design requires respondents to be observed across 7 consecutive waves, leaving too few incident events for reliable estimation.

We then applied additional restrictions. Same-sex couples were excluded due to insufficient power for subgroup analyzes (*n* = 190). We also dropped cases with missing baseline covariates (*n* = 151); although the missing-at-random assumption cannot be verified from observed data alone, our diagnostic checks did not indicate strong evidence against it. We further required continuous participation over time (ie, no skip-return patterns) for at least 7 waves of follow-up from baseline to ensure comparable event-time windows across respondents (*n* = 12 659). We also excluded couples in which both partners experienced a qualifying health shock (*n* = 3469), to avoid conflating spillover effects with the respondent’s own health shock and preserve a clear exposure-outcome structure within the event-study design. After these restrictions, the dataset included 6905 respondents (3313 men, 3592 women) contributing 78 073 person-wave observations (see Figure S1 for analytic sample flow).

Additionally, to improve comparability between exposed and unexposed couples, we implemented 1:1 nearest-neighbor matching in the survey wave immediately preceding shock onset (see Statistical Analysis for more details). This procedure yielded 2084 treated respondents and 2084 matched controls (corresponding to 14 588 treated and 14 588 control person-wave observations), which we then carried forward into the stacked event-study analyzes. The final sample was slightly smaller, at 29 166 person-wave observations, because a small number of observations were dropped during construction of the stacked panel due to missing outcome values in specific waves.

### Measures

#### Exposure

The primary exposure was the incident onset of a major health shock experienced directly by one member of the couple (the index spouse). We then followed the non-index spouse (the partner of the index spouse) to measure spillover effects. Health shocks were defined as the first reported doctor diagnosis of cancer, stroke, or heart disease (including heart attack) reported during follow-up. The shock date was set to the interview wave in which the diagnosis was first reported, and the exposure was treated as absorbing thereafter.

#### Outcomes

The health of the *non-index spouse* (ie, the spouse who did not directly experience the health shock) was tracked biennially. We considered measures of physical, mental, and functional health.

Self-rated health was analyzed as a binary indicator distinguishing poor health (1 = fair/poor) from good health (0 = excellent/very good/good). Chronic disease burden was measured as the count of physician-diagnosed conditions, including heart disease, lung disease, diabetes, hypertension, cancer, stroke, arthritis, and psychiatric conditions. We report results for each specific condition in supplementary analyzes. Physical functioning was measured using standard 5-item activities of daily living (ADL) and instrumental activities of daily living (IADL) difficulty indices, each ranging from 0 to 5. Depressive symptoms were assessed with the 8-item Center for Epidemiologic Studies Depression Scale (CES-D), with positive affect items reverse-coded and summed so that higher values reflect more symptoms.^38^ All outcomes were repeatedly measured across waves.

As secondary outcomes, we examined non-index spouses’ health behaviors and preventive care use. Health behaviors included smoking, coded as current smoker versus former/never, and alcohol use, coded as current drinker versus former/never. Preventive care behaviors, available from wave 3 onward, indicated whether the respondent received a cholesterol blood test or flu shot in the last 2 years. Because these HRS preventive care items were asked in alternating waves, we used the most recent valid response from the nearest administered wave. Since this approach may attenuate short-run changes after a partner’s diagnosis, these findings should be interpreted cautiously. These items capture reported preventive service use in HRS and are not intended to reflect whether care was clinically recommended for a given respondent.

#### Covariates

In our main specification, matching was based on index spouse age, sex, race/ethnicity, and education; non-index spouse age, race/ethnicity, education; and survey wave. The matched outcome models additionally adjusted for a core set of time-varying characteristics measured in the survey wave immediately preceding the event window, approximately 2 years before the shock (ie, 1 wave), to avoid post-treatment bias. These included respondent and spouse age, inflation-adjusted household assets, standardized within each wave, urban versus rural residence, work status, number of children, current smoking status, alcohol use, and physical activity.

In supplementary analyzes, we assessed robustness to broader adjustment for pre-event covariates, including shared risk factors such as baseline health conditions (body mass index, chronic conditions, functional limitations, self-rated health), environmental context (urban locality, state of residence), socioeconomic conditions (household poverty), marital history (marriage duration), healthcare access (government and employer-based insurance coverage), retirement status, survey year fixed effects, and preventive care behaviors. See Appendix S1 for measurement details.

### Statistical analysis

We estimated a stacked event-study design centered on the index spouse’s first diagnosis. This approach captures the timing and trajectory of spousal health changes, rather than summarizing the post-shock period with a single average estimate. For each event, we constructed a balanced 7-wave window (3 waves before and after the diagnosis) and stacked these “sub-experiments” into a pooled panel. The event-time coefficients trace changes in spouses’ outcomes relative to the pre-shock period and identify the average treatment effect on the treated (ATT). We assessed parallel trends by testing whether average pre-treatment effects (4 years) equaled 0 and by visually inspecting event-time plots. The full model specification and stacking algorithm are detailed in Appendix S2.

To account for observable differences between treated and control groups, we implemented 1:1 nearest-neighbor matching^39^ without replacement at the survey wave immediately prior to shock onset and then carried the matched pairs into the stacked sample. Index spouses were matched to control spouses observed in the same pre-event wave. We enforced exact matches on baseline characteristics: index spouse age, sex, race/ethnicity, education; non-index spouse age, race/ethnicity, education; and survey wave. This procedure yielded a comparison group composed of spouses from similarly composed couples who had not experienced a partner shock by the index wave. Balance was assessed using standardized mean differences (Table S1, Figure S2). Standardized differences less than 10% indicated adequate balance.^40^ All analyzes incorporated respondent-level survey weights to account for the sampling design and improve representativeness of the analytic sample.

We conducted several robustness checks. First, to assess whether unobserved differences or pre-diagnosis changes might violate parallel trends, we estimated event-time models with extended pre-treatment leads (*≈*6 years vs 4 years in the main model) and tested for pretrends in covariates that could signal selection—work status/hours, hospitalizations, and medical expenditures.

Second, we varied the event window to *±*1 and *±*3 waves to capture dynamic heterogeneity (short- vs medium-run effects) and assessed the robustness of results to potential attrition and survivorship that grows with longer windows.

Third, we gauged how much results hinged on the treatment operationalization in 2 ways. We first estimated diagnosis-specific models (cancer-only, stroke-only, and heart disease–only) to assess whether pooled estimates are driven by any single diagnosis category. We also contrasted our diagnosis-based definition (first stroke, incident cancer, heart disease) with a broader proxy based on prolonged hospitalization (length of stay *≥*7 days), since some severe health shocks may be better captured by extended hospital stays than by these diagnoses alone.

Fourth, we addressed event-time imprecision from biennial HRS interviews by using available month/year diagnosis information to calculate elapsed time between diagnosis and interview. We then compared more recent shocks (<12 months) with older shocks (12-24 months) to assess whether spillovers were concentrated soon after diagnosis or persisted between interviews.

Fifth, recent work shows that standard stacked difference-in-differences/event-study models can implicitly weight treated and control units unevenly across stacks, so the pooled estimate may not equal the ATT. Following prior work,^41^ we therefore estimated an alternative specification using corrective ATT weights that rebalance the controls across sub-experiments; implementation details are provided in Appendix S2.

Finally, because several sample restrictions may affect generalizability or introduce selection into the analytic sample, we conducted several sensitivity analyzes. These analyzes assessed whether the main findings were robust to including couples in which both partners experienced qualifying health shocks, including same-sex couples, using a less restrictive unbalanced event-study specification that allowed skip-return patterns, and censoring respondents at the time of partnership dissolution rather than continuing follow-up after separation, divorce, or widowhood.

We conducted all analyzes in Stata/MP version 19.5, implementing stacked event-study models (*stackedev* package). The University of California, San Francisco Institutional Review Board determined the study to be exempt from review because there was no direct interaction with human subjects and only de-identified data were analyzed.

## Results

### Descriptive statistics

Table 1 presents summary statistics for the matched sample; pre-matching statistics are available in Appendix S3. The matched sample includes 29 166 person-wave observations, split evenly between treatment and control (14 583 each). Balance was exact or nearly exact on matched characteristics, including age, sex/gender, race/ethnicity, and education. However, the matched sample was highly selected with respect to racial composition (93% White respondents), limiting generalizability to the broader population of older US couples. Remaining differences between treatment and control groups were small but statistically significant for rural residence (33% vs 31%) and work status (14% vs 17%), which were addressed through regression adjustment.

**Table 1:** Descriptive statistics of index and non-index spouses aged 51+ years after matching, HRS, 1992-2020.

	**Overall**	**Control group**	**Treatment group**	**Test for group difference**
**Index spouse (the spouse who experiences a qualifying health shock)**				
*N*	29 166	14 583	14 583	
Age, mean (SD)	72.13 (8.39)	72.13 (8.40)	72.13 (8.39)	1.00
Sex/gender (1 = male, 0 = female), *n* (%)	14 292 (49%)	7146 (49%)	7146 (49%)	1.00
Race/ethnicity, *n* (%)				
1. White/Caucasian	27 124 (93%)	13 562 (93%)	13 562 (93%)	1.00
2. Black/African American	1750 (6%)	875 (6%)	875 (6%)	
3. Other	292 (1%)	146 (1%)	146 (1%)	
Years of education, mean (SD)	12.99 (2.58)	12.99 (2.58)	12.99 (2.58)	1.00
Rural residence (vs urban), *n* (%)	9333 (32%)	4521 (31%)	4812 (33%)	0.03
Working for pay, *n* (%)	4521 (15%)	2479 (17%)	2042 (14%)	<0.00
Number of children, mean (SD)	2.78 (1.57)	2.79 (1.55)	2.77 (1.58)	0.19
**Non-index spouse (partner of the index spouse whose health outcomes are modeled)**				
*N*	26 732	13 366	13 366	
Age, mean (SD)	69.02 (9.40)	69.04 (9.50)	69.00 (9.30)	0.77
Sex/gender (1 = male, 0 = female), *n* (%)	13 016 (49%)	6508 (49%)	6508 (49%)	1.00
Race/ethnicity, *n* (%)				
1. White/Caucasian	17 575 (92%)	8777 (92%)	8798 (92%)	0.25
2. Black/African American	1056 (6%)	516 (5%)	540 (6%)	
3. Other	456 (2%)	244 (3%)	212 (2%)	
Years of education, mean (SD)	12.9 (2.9)	12.9 (2.8)	12.9 (2.9)	0.87
Rural residence (vs urban), *n* (%)	8542 (32%)	4185 (31%)	4357 (33%)	0.08
Working for pay, *n* (%)	5679 (31%)	2841 (31%)	2838 (30%)	0.67
Number of children, mean (SD)	2.78 (1.57)	2.79 (1.55)	2.76 (1.59)	0.20

Abbreviation: HRS, Health and Retirement Study.

(1) Sample is from the HRS, 1992-2020; married or cohabiting opposite-sex couples; 1 respondent per household–wave. (2) The index spouse is the spouse who experiences a qualifying health shock, defined as the first report of cancer, stroke, or heart disease at the index wave. The non-index spouse is the partner of the index spouse whose health outcomes are modeled. (3) “Treatment” refers to matched observations in which the index spouse experiences a qualifying health shock at the index wave; “Control” refers to matched observations in which the index spouse had not yet experienced a qualifying health shock by that wave. (4) Matching: 1:1 nearest-neighbor matching without replacement conducted in the pre-event wave; exact matching on index spouse age, sex, race/ethnicity, and education; non-index spouse age, race/ethnicity, and education; and survey wave. (5) *P*-values come from 2-sample tests (*t*-tests for means; χ2 for proportions).

### Parallel trends assumptions

Figure 1 presents average pre-treatment effects for non-index spouses (the partners whose outcomes we model), computed as the mean of all pre-event lead coefficients from the stacked event-study specification (leads −3 to −1). Visual inspection suggests a mild upward pretrend in chronic conditions (β = .03, 95% CI, 0.00-0.06), although each specific condition does not show clear violations of parallel trends (Figure 2, Table S2). Second, men partnered to women with a diagnosis had slightly fewer ADL/IADL limitations than controls (ADL: β = −.08, 95% CI, −0.14-−0.02; IADL: β = −.06, 95% CI, −0.12- −0.01), indicating modest positive selection and requiring caution in interpreting men’s post-shock estimates. Lastly, men’s CES-D scores showed a modest but non-significant pre-treatment difference (β = .07, 95% CI, −0.04- 0.19).

**Figure 1:**
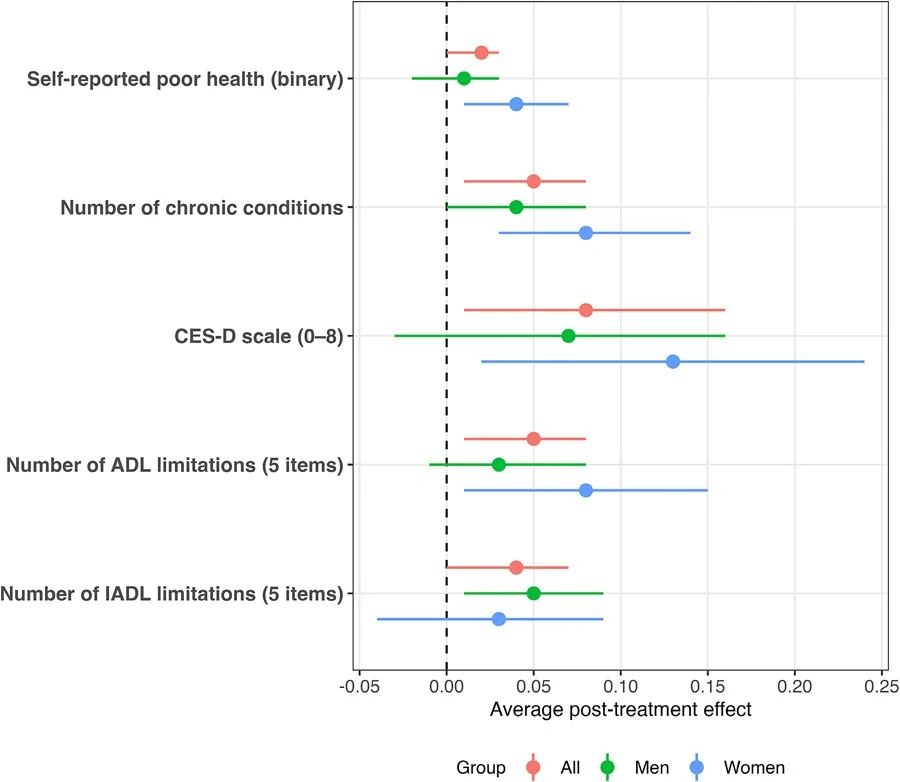
Evaluation of parallel trends: average pre-treatment effects. (1) Sample is from the Health and Retirement Study (HRS), 1992-2020; married or cohabiting opposite-sex couples; 1 respondent per household–wave. (2) Index spouses experienced a health shock (first report of cancer, stroke, or heart disease) at the index wave. (3) Matching: 1:1 nearest-neighbor matching without replacement conducted in the pre-event wave; exact matching on index spouse age, sex, race/ethnicity, and education; non-index spouse age, race/ethnicity, and education; and survey wave. (4) The 8-item Center for Epidemiologic Studies Depression Scale (CES-D) measures depressive symptoms over the past 2 weeks; scores range from 0 (no symptoms) to 8 (more symptoms). (5) Activities of daily living (ADL; 0-5): bathing, dressing, eating, getting in/out of bed, and walking across a room. Instrumental activities of daily living (IADL; 0-5): using the telephone, managing money, taking medications, grocery shopping, and preparing hot meals. Higher scores indicate more limitations. (6) Detailed estimates are provided in Table S2. ^***^P < .01, ^**^P < .05, ^*^P < .10.

**Figure 2:**
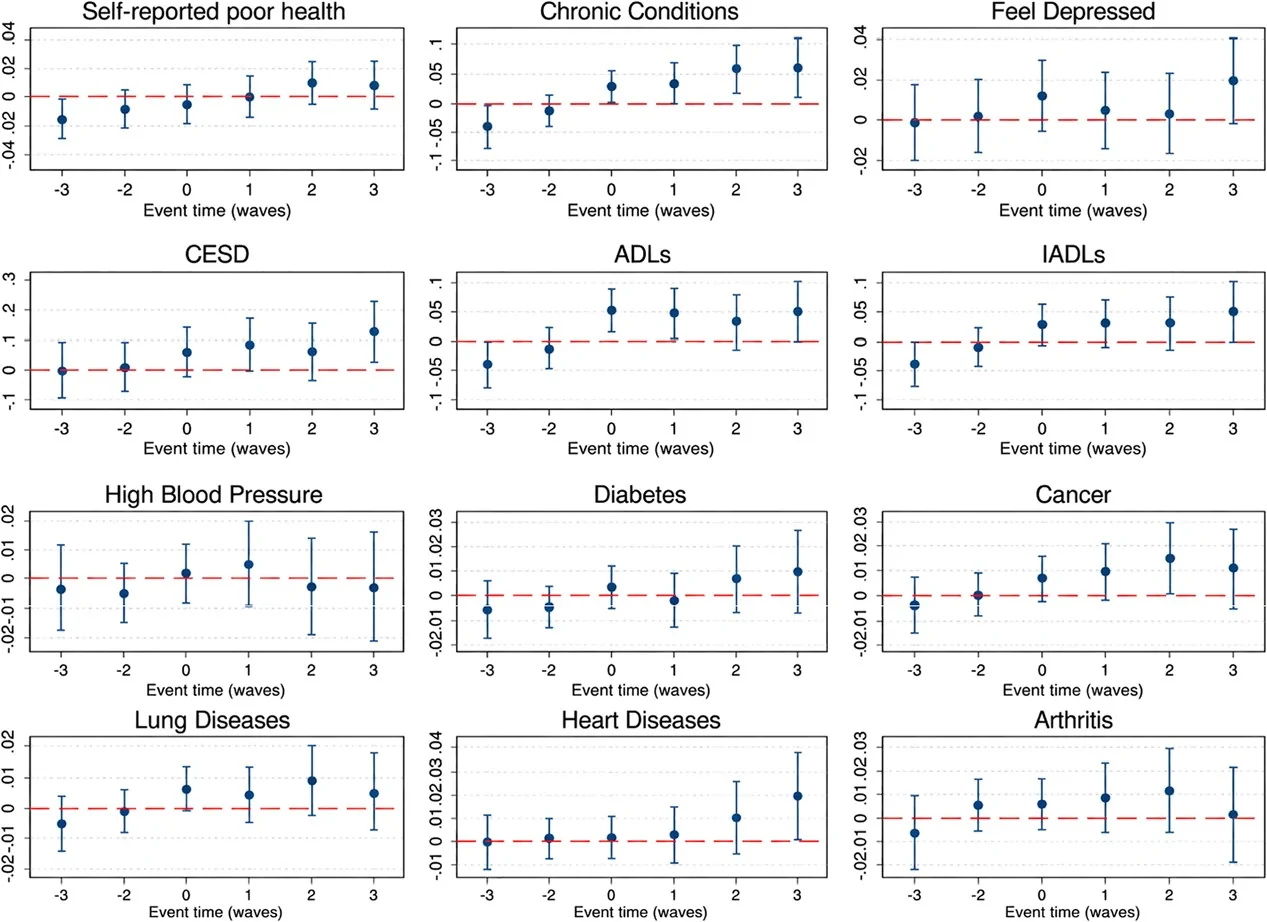
Event-study plots of spouse health outcomes after matching. (1) Sample is from the Health and Retirement Study (HRS), 1992-2020; married or cohabiting opposite-sex couples; 1 respondent per household–wave. (2) Index spouses experienced a health shock (first report of cancer, stroke, or heart disease) at the index wave. (3) Matching: 1:1 nearest-neighbor matching without replacement conducted in the pre-event wave; exact matching on index spouse age, sex, race/ethnicity, and education; non-index spouse age, race/ethnicity, and education; and survey wave. (4) The 8-item Center for Epidemiologic Studies Depression Scale (CES-D) measures depressive symptoms over the past 2 weeks; scores range from 0 (no symptoms) to 8 (more symptoms). (5) Activities of daily living (ADL; 0-5): bathing, dressing, eating, getting in/out of bed, and walking across a room. Instrumental activities of daily living (IADL; 0-5): using the telephone, managing money, taking medications, grocery shopping, and preparing hot meals. Higher scores indicate more limitations. (6) Detailed estimates are provided in Table 2.

For *index spouses* (those who experienced health shocks), event-time plots showed no pre-event drift but a sharp spike in hospitalizations at the diagnosis wave, followed by attenuation over subsequent waves, alongside a drop in self-rated “good” health and a rise in CES-D (see Figure S3). This pattern supports the acuteness of our health-shock definition, with short-run medical intensity and lingering, smaller effects on perceived health and mood.

### Effects of partner health shocks on spouse health outcomes

Table 2 summarizes average post-treatment changes in non-index spouses’ outcomes after a partner’s new diagnosis (see Figure 2 for event-study plots). Following a partner’s health shock, spouses showed evidence of deterioration across several domains, including increases in physician-diagnosed chronic conditions (β = .05, 95% CI, 0.01-0.08), depressive symptoms (CES-D: β = .08, 95% CI, 0.01-0.16), and ADL limitations (β = .05, 95% CI, 0.01-0.08). The chronic-condition estimate, however, should be interpreted cautiously because a mild upward pretrend suggests that part of the increase may reflect continuation of an existing trajectory. Condition-specific estimates were generally imprecise, with only a marginal increase in cancer diagnoses (β = .01, 95% CI, 0.00-0.02). Estimates for IADL limitations were also smaller and less precise (β = .04, 95% CI, 0.00-0.07). Sensitivity analyzes using the extended covariate specification showed a broadly similar pattern, although ADL and IADL estimates were attenuated (Table S3).

**Table 2:** Average post-treatment changes in spouses’ health outcomes after partner’s health shock by sex/gender.

	**Overall**	**Men (partners of women with a health shock)**	**Women (partners of men with a health shock)**
Self-reported poor health (binary)	0.02^c^	0.01	0.04^a^
	(−0.00, 0.03)	(−0.02, 0.03)	(0.01, 0.07)
Number of observations	29 165	14 037	15 128
Number of chronic conditions	0.05^a^	0.04^c^	0.08^a^
	(0.01, 0.08)	(−0.00, 0.08)	(0.03, 0.14)
Number of observations	29 165	14 037	15 128
High blood pressure	0.00	0.00	−0.00
	(−0.01, 0.01)	(−0.02, 0.02)	(−0.03, 0.02)
Number of observations	29 165	14 037	15 128
Diabetes	0.00	0.01	0.01
	(−0.01, 0.02)	(−0.01, 0.02)	(−0.02, 0.02)
Number of observations	29 165	14 037	15 128
Cancer	0.01^b^	0.01	0.02^b^
	(−0.00, 0.02)	(−0.01, 0.02)	(0.003, 0.05)
Number of observations	29 165	14 037	15 128
Lung diseases	0.01	0.01	0.00
	(−0.00, 0.02)	(−0.01, 0.02)	(0.00, 0.05)
Number of observations	29 165	14 037	15 128
Heart diseases	0.01	0.01^c^	0.00
	(−0.00, 0.02)	(−0.00, 0.02)	(−0.01, 0.02)
Number of observations	29 165	14 037	15 128
CES-D scale (0-8)	0.08^b^	0.07	0.13^b^
	(0.01, 0.16)	(−0.03, 0.16)	(0.02, 0.24)
Number of observations	27 439	13 756	13 683
ADL limitations	0.05^b^	0.03	0.08^b^
	(0.01, 0.08)	(−0.01, 0.08)	(0.01, 0.15)
Number of observations	29 163	14 036	15 127
ADL limitations	0.04^b^	0.05^b^	0.03
	(0.00, 0.07)	(0.01, 0.09)	(−0.04, 0.09)
Number of observations	29 163	14 036	15 127

Abbreviations: ADL, activities of daily living; CES-D, Center for Epidemiologic Studies Depression Scale; HRS, Health and Retirement Study; IADL, instrumental activities of daily living.

(1) Sample is from the HRS, 1992-2020; married or cohabiting opposite-sex couples; 1 respondent per household–wave. (2) Index spouses experienced a health shock (first report of cancer, stroke, or heart disease) at the index wave. (3) Matching: 1:1 nearest-neighbor matching without replacement conducted in the pre-event wave; exact matching on index spouse age, sex, race/ethnicity, and education; non-index spouse age, race/ethnicity, and education; and survey wave. (4) The 8-item CES-D measures depressive symptoms over the past 2 weeks; scores range from 0 (no symptoms) to 8 (more symptoms). (5) ADL (0-5): bathing, dressing, eating, getting in/out of bed, and walking across a room. IADL (0-5): using the telephone, managing money, taking medications, grocery shopping, and preparing hot meals. Higher scores indicate more limitations.

a *P* < .01

b *P* < .05

c *P* < .10

### Subgroup analyzes by sex/gender

#### Men (partners of women with a shock)

The evidence suggests little change among men in subjective health, depressive symptoms, or chronic conditions. However, there was some evidence of worsening functional capacity, concentrated in IADLs (β = .05, 95% CI, 0.01-0.09), although this estimate should be interpreted cautiously because men in the treatment group had fewer ADL/IADL limitations than controls at baseline, so some of the post-shock increase may reflect convergence rather than the partner’s health shock alone.

#### Women (partners of men with a shock)

Women showed more consistent post-shock deterioration across health domains than men. Women reported more self-rated poor health (β = .04, 95% CI, 0.01-0.07) and more chronic conditions (β = .08, 95% CI, 0.03-0.14). Women also exhibited more depressive symptoms (β = .13, 95% CI, 0.02-0.24) and ADL limitations (β = .08, 95% CI, 0.01-0.15).

Formal interaction tests indicated that post-shock changes differed by sex for self-reported health, the number of chronic conditions, and depressive symptoms, with weaker evidence of sex differences for ADL and IADL limitations (Table S4).

### Subgroup analyzes by socioeconomic status

Stratifying by the non-index spouse’s education (partners of respondents with a health shock) suggests that impacts were concentrated among those with lower education and household assets (Figure 3, Tables S5 and S6). Among spouses with a high school education or less, depressive symptoms increased (CES-D: β = .13, 95% CI, 0.03-0.23), whereas the estimates for self-rated poor health (β = .03, 95% CI, −0.00- 0.05) and the number of chronic conditions (β = .04, 95% CI, −0.01-0.09) were not statistically different from 0. Functional limitations also trended upward, although the ADL and IADL estimates were modest and imprecise (ADL: β = .04, 95% CI, 0.00-0.09; IADL: β = .04, 95% CI, 0.00-0.09). For spouses with some college or more, we observed only a small, marginal increase in chronic conditions (β = .05, 95% CI, 0.00-0.10).

**Figure 3:**
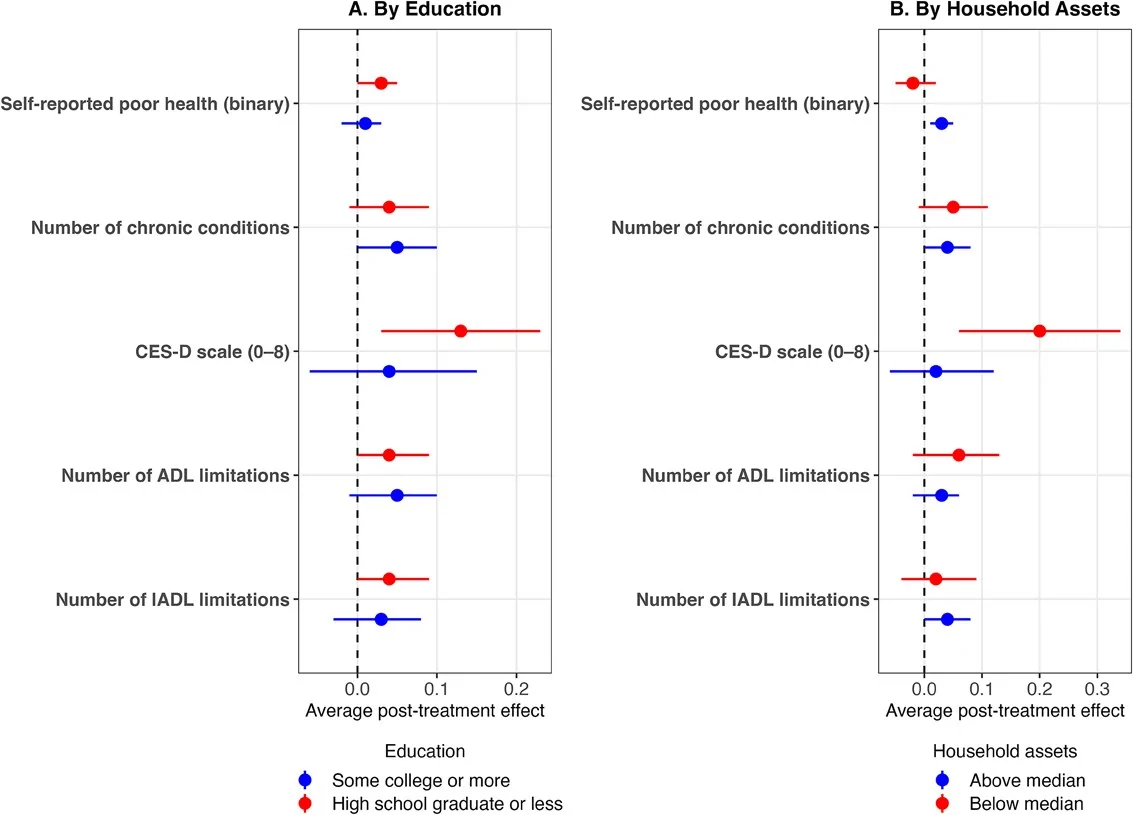
Average post-treatment changes in spouses’ health outcomes after partner’s health shock by education and household assets. (1) Sample is from the Health and Retirement Study (HRS), 1992-2020; married or cohabiting opposite-sex couples; 1 respondent per household–wave. (2) Index spouses experienced a health shock (first report of cancer, stroke, or heart disease) at the index wave. (3) Matching: 1:1 nearest-neighbor matching without replacement conducted in the pre-event wave; exact matching on index spouse age, sex, race/ethnicity, and education; non-index spouse age, race/ethnicity, and education; and survey wave. (4) The 8-item Center for Epidemiologic Studies Depression Scale (CES-D) measures depressive symptoms over the past 2 weeks; scores range from 0 (no symptoms) to 8 (more symptoms). (5) Activities of daily living (ADL; 0-5): bathing, dressing, eating, getting in/out of bed, and walking across a room. Instrumental activities of daily living (IADL; 0-5): using the telephone, managing money, taking medications, grocery shopping, and preparing hot meals. Higher scores indicate more limitations. (6) Detailed estimates are provided in Tables S5–S6. ^***^P < .01, ^**^P < .05, ^*^P < .10.

Among below-median asset households, depressive symptoms increased significantly (β = .20, 95% CI, 0.06-0.34), but evidence for an increase in chronic conditions was weaker (β = .05, 95% CI, −0.01-0.11). In contrast, in above-median asset households, spouses reported a modest increase in self-rated poor health (β = .03, 95% CI, 0.01-0.05), while the estimates for chronic conditions (β = .04, 95% CI, 0.00-0.08) and IADL limitations (β = .04, 95% CI, 0.00-0.08) were less precise. Overall, the pattern suggests mental health vulnerabilities were concentrated among spouses with fewer-assets, whereas higher-asset spouses exhibited subtle deteriorations in perceived health.

### Effects of partner health shocks on spouse health behaviors

We find little evidence that partner shocks changed spouses’ smoking, drinking, or preventive care use (Figure 4, Tables S7 and S8). Adjusting for these potential mediators did not materially change the estimated effects of partner health shocks in our main analyzes (Table S9).

**Figure 4:**
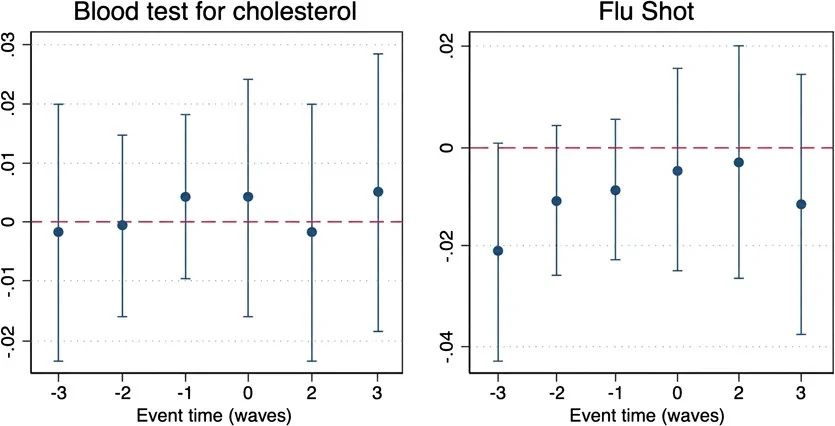
Event-study plots of spouse preventive health behaviors. (1) Sample is from the Health and Retirement Study (HRS), 1992-2020; married or cohabiting opposite-sex couples; 1 respondent per household–wave. (2) Index spouses experienced a health shock (first report of cancer, stroke, or heart disease) at the index wave. (3) Matching: 1:1 nearest-neighbor matching without replacement conducted in the pre-event wave; exact matching on index spouse age, sex, race/ethnicity, and education; non-index spouse age, race/ethnicity, and education; and survey wave. (4) All outcomes are binary indicators (1 = yes) measured at interview. (5) Detailed estimates are provided in Table S8. ^***^P < .01, ^**^P < .05, ^*^P < .10.

### Robustness tests

In a series of robustness checks (Tables S10-S16, Appendices S4 and S5), we found that our main results were stable across alternative specifications. Event-time models with multiple leads showed little evidence of anticipatory effects or differential pretrends, even when we examined additional indicators that could signal selection (eg, employment, hospitalization, and medical spending). Estimates were also highly similar when we varied the length of the post-shock window (1-, 2-, and 3-wave follow-ups). Diagnosis-specific models (cancer only, stroke only, and heart disease only) yielded point estimates that were generally consistent in direction with the pooled estimates for key outcomes, particularly increases in functional limitations, depressive symptoms, and worse self-rated health. However, precision decreased in these models overall, reflecting smaller sample sizes. Using a broader treatment definition based on prolonged hospitalization yielded effects in the same direction but somewhat larger and less precisely estimated. Using month/year diagnosis information where available, results were similar when distinguishing more recent shocks (<12 months before interview) from older shocks (12-24 months). In addition, applying corrective ATT weights for stacked event studies had negligible impact on the magnitude or interpretation of the estimates. Finally, the main pattern of results was generally similar across alternative analytic samples, including specifications that included couples in which both partners experienced shocks and same-sex couples, allowed skip-return patterns, and censored respondents at partnership dissolution (Appendix S4). However, the CES-D estimate was attenuated when dual-shock couples were included (*β* = .08 to *β* = .01). This may reflect the difficulty of isolating partner spillover effects and aligning event timing in couples where both partners experienced shocks. For example, if the respondent’s own health decline occurred before or near the partner’s diagnosis, depressive symptoms may have already increased before the partner-shock event window, leaving less detectable change afterward.

## Discussion

Major health shocks—such as first diagnoses of cancer, stroke, or heart disease—are common, sudden, and economically and clinically consequential for patients and their families. While international evidence documents substantial within-household health spillovers, US research has largely emphasized labor market and financial outcomes or has relied on caregiver-selected samples that cannot recover population effects. This study addresses that gap by estimating the health consequences for spouses in the United States following a partner’s acute diagnosis, and by characterizing how these effects vary by gender and socioeconomic status. Using rich longitudinal data and a matched, stacked event-study design centered on plausibly exogenous first diagnoses, we recover dynamic average treatment effects and provide evidence that reframes major illness as a household event, informing targeted supports for spouses most at risk.

Our findings indicate that a partner’s first major diagnosis reverberates beyond the patient, producing measurable and persistent health burdens for spouses. Spouses experienced modest declines in self-rated health (a 2-percentage-point higher probability of reporting poor health), small increases in diagnosed morbidity (≈2%-3% of the baseline), higher depressive symptoms (≈7%-8% of the baseline), and worsening functional capacity (≈15%-20% of the baseline), on average over the 6 years following diagnosis, relative to matched controls.

Although prior work differs in how “health shocks” are defined and is often set in health systems with more universal coverage and long-term care supports than the US, our results are consistent with growing quasi-experimental evidence from the Netherlands,^3^ Sweden,^6^ and across multiple European countries,^35^ documenting broader morbidity and mental health declines after a partner’s health shock, as well as observational findings of elevated spousal mortality following hospitalizations, especially for cognitive/psychiatric and frailty-type events in the US.^4^^,^^42^

These effects are not well explained by short-run changes in routine preventive care or caregiving behaviors. This pattern aligns with recent studies documenting worsened mental health without clear behavioral change pathways,^3^^,^^35^ and in some cases, including reports of increased exercise and reduced drinking after a partner’s diagnosis,^43^ as well as higher medical expenditure on preventive care.^44^ Caregiving experiences are heterogeneous: many caregivers report little or no strain, and caregiving has been linked to lower mortality risk in several population-based studies.^45^ Our evidence suggests that the harm to spouses may reflect a broader relational stress response, rather than caregiving burden alone. A partner’s serious diagnosis can generate sustained emotional vigilance, worry, and uncertainty even before or apart from intensive hands-on care.^46^^,^^47^ These chronic stress and resource-constraint pathways may in turn contribute to mental health and functional declines through cumulative physiological wear and tear.

Furthermore, the distribution of these spillovers is uneven. Women partnered to men experiencing a health shock showed broader and more pronounced deterioration than men partnered to women, a pattern that may be consistent with gendered caregiving roles and related time and stress demands.^48^^‑^^50^ Socioeconomic resources also matter: spouses with less education and fewer assets displayed the clearest mental health and functional losses, suggesting that access to resources and caregiving roles may shape vulnerability. Integrating spouses into post-diagnosis care planning, connecting families to supportive services early, and targeting outreach to lower-resource households may help mitigate these downstream health costs and strengthen recovery for both members of the couple.^51^^,^^52^

This study has several limitations. First, even after matching and pretrend checks, residual bias may remain: common-fate processes, early diagnostic work-ups, or prodromal decline may precede the recorded diagnosis, and unobserved factors (eg, latent health risks or relationship quality) may still confound estimated spousal effects. Second, linked administrative or clinical data with exact diagnosis dates and more frequent outcome measurement could support a more precise survival or time-to-event framework in future work, but this would require a substantively different design from the present study. Third, we focus on the first *observed* acute diagnosis, which may not coincide with true clinical onset and does not capture the cumulative impact of subsequent shocks, likely biasing short-run estimates downward toward 0.

Finally, severe illness and caregiving burden can increase dropout or mortality for both partners; if attrition is selective, our longer-run estimates may understate the true magnitude of spillover effects. Future research using linked clinical or administrative data and richer caregiving measures could sharpen event timing, clarify mechanisms, and identify at-risk couples.

## Supplementary Material

Web_Material_kwag178

## Data Availability

The data used in this study are publicly available from the Health and Retirement Study (HRS; https://hrsdata.isr.umich.edu/data-products/rand). The code required to replicate the results is available on GitHub (https://github.com/arlee27/Partner-Health-After-Spousal-Health-Shocks).
