# Supplementary material for: Health consequences of spousal health shocks: evidence from a matched event study in the United States
Source: Am J Epidemiol. 2026 Jul 24;195(10):2518–27. doi: 10.1093/aje/kwag178 (PMC13642367; doi:10.1093/aje/kwag178)
Supplement: Supplementary file 1 [file web_material_kwag178.zip › Web_Material_kwag178.pdf]

Supplemental Digital Content

Article Title: Health Consequences of Spousal Health Shocks: Evidence from a Matched Event Study in the United States

Authors

Ah-Reum Lee*, Department of Epidemiology and Biostatistics, University of California, San Francisco, USA (ahreum.lee@ucsf.edu)

Ashwin Kotwal, Division of Geriatrics, University of California, San Francisco (ashwin.kotwal@ucsf.edu)

Jacqueline M. Torres, Department of Epidemiology and Biostatistics, University of California, San Francisco, USA (jacqueline.torres@ucsf.edu)

*Corresponding author

List of Supplementary Materials

Figure S1 Study Sample Selection and Exclusions
Figure S2 Difference in Baseline Characteristics between Households with and without Spouse Health Shocks: Before and After matching
Figure S3. Event-Study Trajectories for Index Spouse Hospitalization and Health Around First Major Diagnosis

Table S1 Matching Diagnostics
Table S2 Evaluation of Parallel Trends: Average Pre-Treatment Effects After Matching
Table S3 Average Post-Treatment Changes in Spouses' Health Outcomes After a Partner's Health Shock With an Extended Covariate Set
Table S4 Formal Tests of Sex Differences in Post-Period Spillover Effects
Table S5 Average Post-Treatment Changes in Spouses' Health Outcomes After Partner's Health Shock by Non-Index Spouses' Educational Attainment
Table S6 Average Post-Treatment Changes in Spouses' Health Outcomes After Partner's Health Shock by Household Asset
Table S7 Average Post-Treatment Changes in Spouses' Health Behaviors After Partner's Health Shock
Table S8 Average Post-Treatment Changes in Spouses' Preventive Behaviors After Partner's Health Shock: Averages over Varying Post-Shock Windows
Table S9 Results Adjusted for Additional Mediators: Preventive Care Behaviors (Available From Wave 3 Onward)
Table S10 Average Pre-Treatment Effects with Three-Wave Leads (~6 Years)
Table S11 Average Pre- and Post-Treatment Effects on Selection-Related Outcomes
Table S12 Average Post-Treatment Changes in Spouses' Health Outcomes After Partner's Health Shock: Estimates by Individual Diagnosis
Table S13 Average Post-Treatment Changes in Spouses' Health Outcomes After Partner's Health Shock: Averages over Varying Post-Shock Windows
Table S14 Main Definition (First Cancer/Stroke/Heart Disease) vs. Broad Definition (Hospital Stay ≥7 Days)
Table S15 Average Post-Treatment Changes in Spouses' Health Outcomes by Time Since Partner's Health Shock
Table S16 Spousal Health Outcomes After Partner's Health Shock: Corrective ATT Weights vs. Unweighted Estimates

Appendix S1. Definitions and Measurement of Outcomes and Covariates
Appendix S2. Models
Appendix S3. Pre-matching Statistics
Appendix S4. Sensitivity Analyses for Sample Restrictions
Appendix S5. Robustness tests

Figure S1 Study Sample Selection and Exclusions

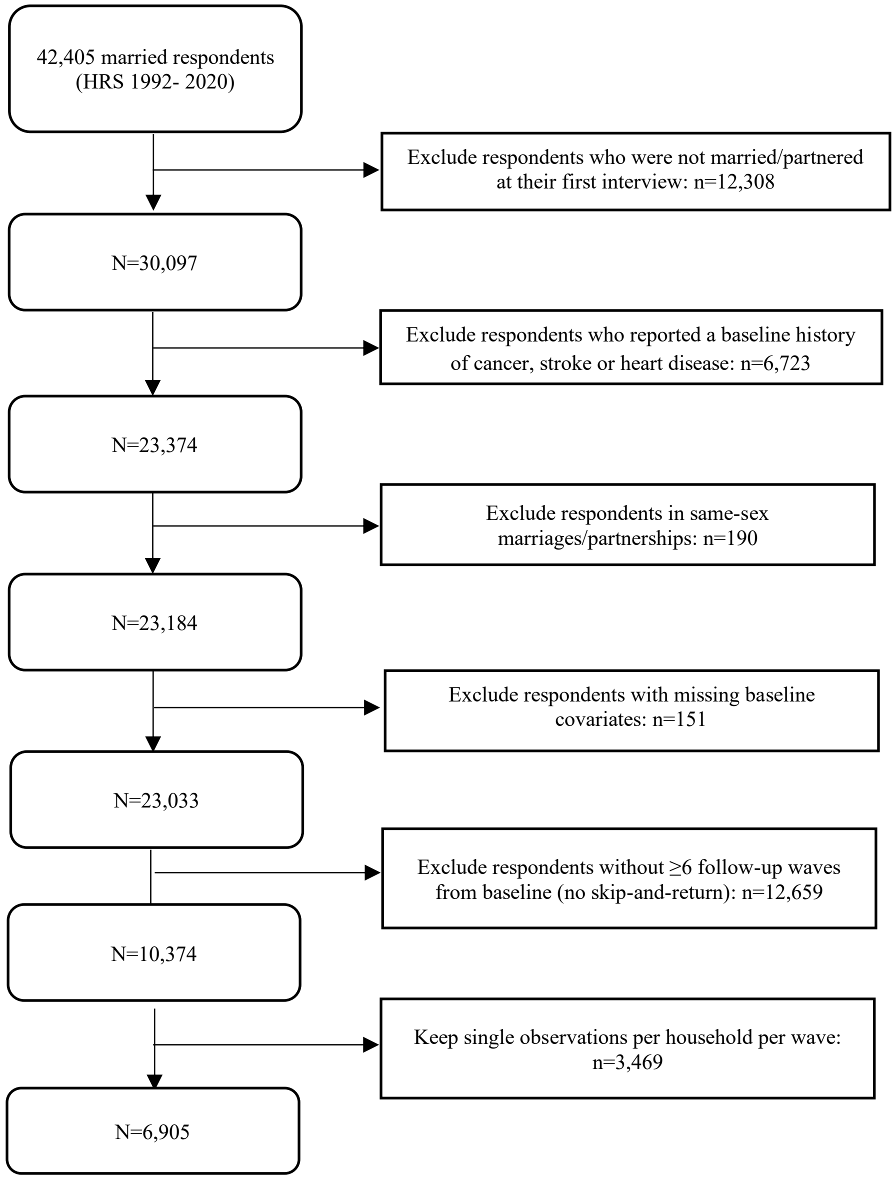

Figure S2 Difference in Baseline Characteristics between Households with and without Spouse Health Shocks: Before and After matching

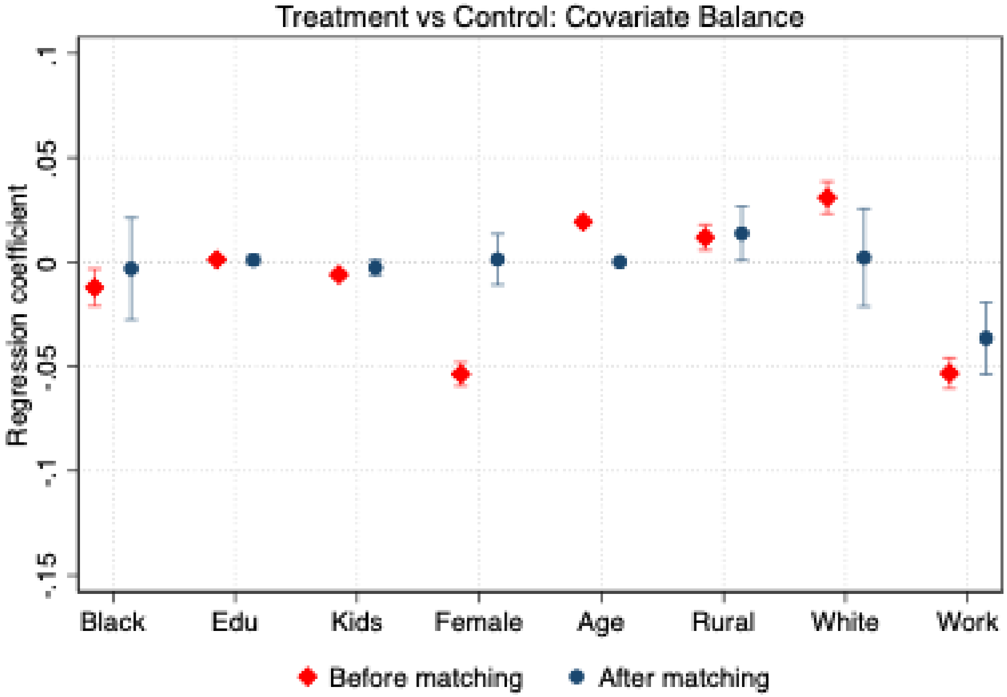

Sources/Notes. 1) Sample is from the Health and Retirement Study (HRS), 1992–2020; opposite-sex married/cohabiting couples; one respondent per household–wave. 2) "Treatment" = index spouses experience a health shock (first report of cancer, stroke, or heart disease) at the index wave; "Control" = index spouses who had not experienced a qualifying shock by that wave (never or not yet). 3) Points show OLS coefficients and 95% CIs from separate regressions of each characteristic on an indicator for partner health shock. Panel "Before" uses the full eligible sample; panel "After" uses 1:1 nearest-neighbor matched pairs (no replacement) constructed in the pre-event wave (r–1) with the exact-match criteria described in **Table S1** notes. A coefficient of 0 indicates balance; positive (negative) values indicate higher (lower) means in the treatment group relative to controls.

Figure S3. Event-Study Trajectories for Index Spouse Hospitalization and Health Around First Major Diagnosis

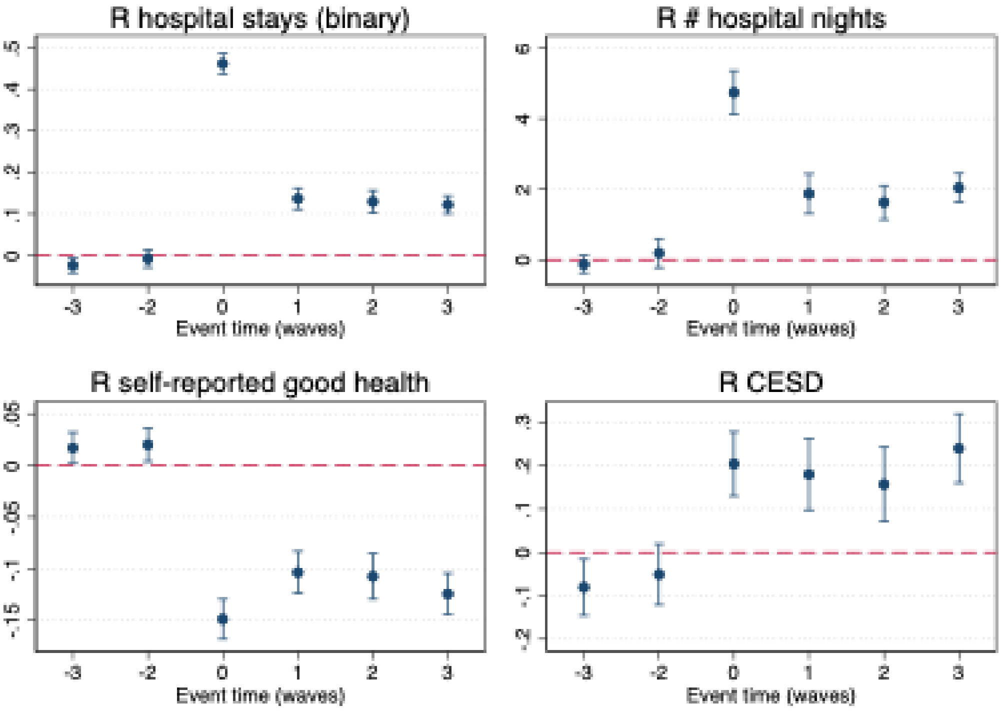

Notes. The event-study plots for the index spouse corroborate the acuteness of our shock definition. Hospitalization incidence and nights are flat in the pre-period and surge sharply at $r = 0$, then decline toward baseline within the next one to two waves – exactly the profile expected for an acute medical event. In parallel, self-reported "good" health drops at the event and remains modestly lower thereafter, while CES-D shows a contemporaneous rise with some persistence. The combination of no pretrends, a discrete spike at onset, and attenuation post-shock indicates that the first diagnosis (cancer, stroke, or heart disease) captures an acute episode with short-run medical intensity and lingering, but smaller, consequences for perceived health and mood.

Table S1 Matching Diagnostics

	Mean		
	**Treated**	**Untreated**	**StdDif**
**Index spouse characteristics**			
Age	72.13	72.13	0.00
Gender	0.49	0.49	0.00
Black/African American	0.06	0.06	0.00
Other race/ethnicity	0.01	0.01	0.00
Years of education	12.99	12.99	0.00
Work status	2.77	2.79	-0.01
Rural	0.33	0.31	-0.04
**Non-index spouse characteristics**			
Age	69.02	69.02	0.00
Black/African American	0.06	0.05	-0.02
Other race/ethnicity	0.02	0.03	0.03
Years of education	12.90	12.91	0.00

Sources/Notes. 1) Sample is from the Health and Retirement Study (HRS), 1992–2020; opposite-sex married/cohabiting couples; one respondent per household–wave. 2) "Treatment" = index spouses experience a health shock (first report of cancer, stroke, or heart disease) at the index wave; "Control" = index spouses who had not experienced a qualifying shock by that wave (never or not yet). 3) Matching: 1:1 nearest-neighbor matching without replacement conducted in the pre-event wave; exact matching on index spouse age, sex, race/ethnicity, education; non-index spouse age and education; and survey wave. 4) "Mean" columns report post-matching means; for binary variables (e.g., Gender, Black/African American, Rural), values are proportions. "StdDif" is the standardized mean (or variance) difference between treated and untreated groups. Values with |StdDif|<0.10 indicate negligible imbalance; values near 0.00/1.00 reflect near-exact balance on means/variances by design.

Table S1 Evaluation of Parallel Trends: Average Pre-Treatment Effects After Matching

	All	Women (partners of men with a health shock)	Men (partners of women with a health shock)
**Self-reported poor health (binary)**	-0.01	-0.02	0.01
	(-0.03, 0.01)	(-0.04, 0.00)	(-0.02, 0.04)
**Number of observations**	29,165	14,037	15,128
**Number of chronic conditions**	0.03*	0.03	0.05
	(0.00, 0.06)	(0.00, 0.07)	(0.02, 0.08)
**Number of observations**	29,165	14,037	15,128
**CES-D scale (0-8)**	0.01	-0.04	0.07
	(-0.07, 0.08)	(-0.14, 0.06)	(-0.04, 0.19)
**Number of observations**	27,439	13,756	13,683
**Number of ADL limitations (5 items)**	-0.03	0.00	-0.08***
	(-0.06, 0.01)	(-0.04, 0.04)	(-0.14, -0.02)
**Number of observations**	29,163	14,036	15,127
**Number of IADL limitations (5 items)**	-0.02	-0.01	-0.06**
	(-0.05, 0.01)	(-0.04, 0.03)	(-0.12, -0.01)
**Number of observations**	29,163	14,036	15,127

Sources/Notes. 1) Sample is from the Health and Retirement Study (HRS), 1992–2020; opposite-sex married/cohabiting couples; one respondent per household–wave. 2) Index spouses experience a health shock (first report of cancer, stroke, or heart disease) at the index wave. 3) Matching: 1:1 nearest-neighbor matching without replacement conducted in the pre-event wave; exact matching on index spouse age, sex, race/ethnicity, education; non-index spouse age and education; and survey wave. 4) The 8-item CES-D measures depressive symptoms over the past two weeks; scores range from 0 (no symptoms) to 8 (more symptoms). 5) ADL (0–5): bathing, dressing, eating, getting in/out of bed, and walking across a room. IADL (0–5): using the telephone, managing money, taking medications, grocery shopping, and preparing hot meals. Higher scores indicate more limitations 6) The reported coefficient for each outcome is the Average Pre-Treatment Effect, computed as the mean of all pre-event lead coefficients from the stacked event-study specification. *** $p<0.01$, ** $p<0.05$, * $p<0.10$.

Table S3 Average Post-Treatment Changes in Spouses' Health Outcomes After a Partner's Health Shock with an Extended Covariate Set

	Main specification	Extended covariates
**Self-reported poor health (binary)**	0.02*	0.02*
	(-0.00, 0.03)	(0.01, 0.03)
**Number of observations**	29,165	29,115
**Number of chronic conditions**	0.05***	0.05***
	(0.01, 0.08)	(0.02, 0.08)
**Number of observations**	29,165	29,115
**High blood pressure**	0.00	0.00
	(-0.01, 0.01)	(-0.02, 0.02)
**Number of observations**	29,165	29,115
**Diabetes**	0.00	0.01
	(-0.01, 0.02)	(-0.00, 0.03)
**Number of observations**	29,165	29,115
**Cancer**	0.01**	0.01*
	(-0.00, 0.02)	(0.00, 0.02)
**Number of observations**	29,165	29,115
**Lung diseases**	0.01	0.00
	(-0.00, 0.02)	(-0.01, 0.02)
**Number of observations**	29,165	29,115
**Heart diseases**	0.01	0.01
	(-0.00, 0.02)	(-0.01, 0.03)
**Number of observations**	29,165	29,115
**CES-D scale (0-8)**	0.08**	0.09***
	(0.01, 0.16)	(0.03, 0.16)
**Number of observations**	27,439	27,289
**ADL limitations**	0.05**	0.03**
	(0.01, 0.08)	(0.01, 0.07)
**Number of observations**	29,163	28,513
**IADL limitations**	0.04**	0.03
	(0.00, 0.07)	(-0.03, 0.10)
**Number of observations**	29,163	28,513

Notes. 1) Sample is from the Health and Retirement Study (HRS), 1992–2020; opposite-sex married/cohabiting couples; one respondent per household-wave. 2) Index spouses experience a health shock (first report of cancer, stroke, or heart disease) at the index wave. 3) Matching: 1:1 nearest-neighbor matching without replacement conducted in the pre-event wave; exact matching on index spouse age, sex, race/ethnicity, education; non-index spouse age and education; and survey wave. 4) The 8-item CES-D measures depressive symptoms over the past two weeks; scores range from 0 (no symptoms) to 8 (more symptoms). 5) ADL (0–5): bathing, dressing, eating, getting in/out of bed, and walking across a room. IADL (0–5): using the telephone, managing money, taking medications, grocery shopping, and preparing hot meals. Higher scores indicate more limitations. *** *p<0.01,* ** *p<0.05,* * *p<0.10.*

Table S4 Formal Tests of Sex Differences in Post-Period Spillover Effects

Outcome	F-test	p-values
Self-reported poor health	3.35	0.01
Number of chronic conditions	3.30	0.01
CES-D scale (0–8)	2.93	0.02
Number of ADL limitations (5 items)	2.19	0.067
Number of IADL limitations (5 items)	1.95	0.1

Sources/Notes. Sample is from the Health and Retirement Study (HRS), 1992–2020; opposite-sex married/cohabiting couples; one respondent per household-wave. 2) Matching: 1:1 nearest-neighbor matching without replacement conducted in the pre-event wave; exact matching on index spouse age, sex, race/ethnicity, education; non-index spouse age, race/ethnicity, education; and survey waves. 3) Interaction tests evaluate whether post-period effects of a partner's health shock differ by sex (women vs men) by interacting the post-period event-time indicators with an indicator for female spouses. Reported statistics are joint F-tests for the post-period interaction terms. *** *p<0.01*, ** *p<0.05*, * *p<0.10.*

Table S5 Average Post-Treatment Changes in Spouses' Health Outcomes After Partner's Health Shock by Non-Index Spouses' Educational Attainment

	High School Graduate or Less	Some College or More
**Self-reported poor health (binary)**	0.03**	0.01
	(0.00, 0.05)	(-0.02, 0.03)
**Number of observations**	13,376	15,789
**Number of chronic conditions**	0.04*	0.05**
	(-0.01, 0.09)	(0.00, 0.10)
**Number of observations**	13,376	15,789
**CES-D scale (0-8)**	0.13**	0.04
	(0.03, 0.23)	(-0.06, 0.15)
**Number of observations**	12,704	16,461
**Number of ADL limitations (5 items)**	0.04*	0.05
	(-0.00, 0.09)	(-0.01, 0.10)
**Number of observations**	13,376	15,787
**Number of IADL limitations (5 items)**	0.04*	0.03
	(-0.00, 0.09)	(-0.03, 0.08)
**Number of observations**	13,376	15,787

Sources/Notes. 1) Sample is from the Health and Retirement Study (HRS), 1992–2020; opposite-sex married/cohabiting couples; one respondent per household-wave. 2) Index spouses experience a health shock (first report of cancer, stroke, or heart disease) at the index wave; non-index spouses are their partners, i.e., spouses whose partner experiences the health shock. 3) Matching: 1:1 nearest-neighbor matching without replacement conducted in the pre-event wave; exact matching on index spouse age, sex, race/ethnicity, education; non-index spouse age and education; and survey wave. 4) The 8-item CES-D measures depressive symptoms over the past two weeks; scores range from 0 (no symptoms) to 8 (more symptoms). 5) ADL (0–5): bathing, dressing, eating, getting in/out of bed, and walking across a room. IADL (0–5): using the telephone, managing money, taking medications, grocery shopping, and preparing hot meals. Higher scores indicate more limitations. *** $p<0.01$, ** $p<0.05$, * $p<0.10$.

Table S6 Average Post-Treatment Changes in Spouses' Health Outcomes After Partner's Health Shock by Household Asset

	Below Median	Above Median
**Self-reported poor health (binary)**	-0.02	0.03***
	(-0.05, 0.02)	(0.01, 0.05)
**Number of observations**	12,549	16,616
**Number of chronic conditions**	0.05*	0.04*
	(-0.01, 0.11)	(0.00, 0.08)
**Number of observations**	12,549	16,616
**CES-D scale (0-8)**	0.20***	0.02
	(0.06, 0.34)	(-0.06, 0.12)
**Number of observations**	13,940	18,996
**Number of ADL limitations (5 items)**	0.06	0.03
	(-0.02, 0.13)	(-0.02, 0.06)
**Number of observations**	12,547	16,616
**Number of IADL limitations (5 items)**	0.02	0.04*
	(-0.04, 0.09)	(0.00, 0.08)
**Number of observations**	12,547	16,616

Sources/Notes. 1) Sample is from the Health and Retirement Study (HRS), 1992–2020; opposite-sex married/cohabiting couples; one respondent per household-wave. 2) Index spouses experience a health shock (first report of cancer, stroke, or heart disease) at the index wave. 3) Matching: 1:1 nearest-neighbor matching without replacement conducted in the pre-event wave; exact matching on index spouse age, sex, race/ethnicity, education; non-index spouse age and education; and survey wave. 4) The 8-item CES-D measures depressive symptoms over the past two weeks; scores range from 0 (no symptoms) to 8 (more symptoms). 5) ADL (0–5): bathing, dressing, eating, getting in/out of bed, and walking across a room. IADL (0–5): using the telephone, managing money, taking medications, grocery shopping, and preparing hot meals. Higher scores indicate more limitations. *** *p<0.01,* ** *p<0.05,* * *p<0.10.*

Table S7 Average Post-Treatment Changes in Spouses' Health Behaviors After Partner's Health Shock

	ATT
**Drink now**	0.00
	(-0.04, 0.04)
**Number of observations**	29,177
**Smoke now**	0.00
	(-0.01, 0.01)
**Number of observations**	29,881
**Blood test for cholesterol**	0.00
	(-0.01, 0.02)
**Number of observations**	28,954
**Flu shot**	-0.01
	(-0.03, 0.01)
**Number of observations**	28,990

Sources/Notes. 1) Sample is from the Health and Retirement Study (HRS), 1992–2020; opposite-sex married/cohabiting couples; one respondent per household-wave. 2) Index spouses experience a health shock (first report of cancer, stroke, or heart disease) at the index wave. 3) Matching: 1:1 nearest-neighbor matching without replacement conducted in the pre-event wave; exact matching on index spouse age, sex, race/ethnicity, education; non-index spouse age and education; and survey wave. 4) ATT = average treatment effect on the treated; estimates represent the average difference in outcomes between treated spouses and their matched controls. 5) All outcomes are binary indicators (1=yes) measured at interview. Drink now = currently drinks alcohol. Smoke now = currently smokes. Preventive care items (blood test for cholesterol, flu shot) indicate whether the respondent reports receiving the service since the last survey wave. *** $p<0.01$, ** $p<0.05$, * $p<0.10$.

Table S8 Average Post-Treatment Changes in Spouses' Preventive Behaviors After Partner's Health Shock: Averages over Varying Post-Shock Windows

**Follow-up periods**	**1 wave (≈2 years)**	**2 waves (≈ 4 years)**	**3 waves (≈ 6 years; main model)**
**Blood test for cholesterol**	0.01<br>(-0.01, 0.02)	0.00<br>(-0.01, 0.02)	0.00<br>(-0.01, 0.02)
**Number of observations**	28,844	28,844	28,954
**Flu shot**	-0.01<br>(-0.02, 0.01)	-0.006<br>(-0.02, 0.01)	-0.007<br>(-0.03, 0.01)
**Number of observations**	28,894	28,894	28,990

Sources/Notes. 1) Sample is from the Health and Retirement Study (HRS), 1992–2020; opposite-sex married/cohabiting couples; one respondent per household-wave. 2) Index spouses experience a health shock (first report of cancer, stroke, or heart disease) at the index wave. 3) Matching: 1:1 nearest-neighbor matching without replacement conducted in the pre-event wave; exact matching on index spouse age, sex, race/ethnicity, education; non-index spouse age and education; and survey wave. 4) ATT = average treatment effect on the treated; estimates represent the average difference in outcomes between treated spouses and their matched controls. *** *p<0.01,* ** *p<0.05,* * *p<0.10.*

.

Table S9 Results Adjusted for Additional Mediators: Preventive Care Behaviors (Available From Wave 3 Onward)

	All	Men (partners of women with a health shock)	Women (partners of men with a health shock)
**Self-reported poor health (binary)**	0.02**	0.01	0.04***
	(-0.00, 0.03)	(-0.02, 0.03)	(0.01, 0.07)
**Number of observations**	29,004	14,037	15,128
**Number of chronic conditions**	0.05***	0.04*	0.08***
	(0.01, 0.08)	(-0.00, 0.08)	(0.02, 0.14)
**Number of observations**	29,004	14,037	15,128
**CES-D scale**	0.08**	0.06	0.13**
	(0.01, 0.16)	(-0.03, 0.16)	(0.03, 0.24)
**Number of observations**	27,298	13,756	13,683
**Feeling depressed**	0.01	0.01	0.02*
	(-0.01, 0.03)	(-0.01, 0.03)	(-0.00, 0.04)
**Number of observations**	27,312	13,756	13,697
**Number of ADL limitations (5 items)**	0.05***	0.03	0.08**
	(0.01, 0.08)	(-0.01, 0.08)	(0.02, 0.15)
**Number of observations**	29,002	14,036	15,127
**Number of IADL limitations (5 items)**	0.04**	0.05**	0.04
	(0.00, 0.07)	(0.01, 0.09)	(-0.03, 0.10)
**Number of observations**	29,002	14,036	15,127

Sources/Notes. 1) Sample is from the Health and Retirement Study (HRS), 1992–2020; opposite-sex married/cohabiting couples; one respondent per household-wave. 2) Matching: 1:1 nearest-neighbor matching without replacement conducted in the pre-event wave; exact matching on index spouse age, sex, race/ethnicity, education; non-index spouse age, race/ethnicity, education; and survey waves. 3) ATT = average treatment effect on the treated; estimates represent the average difference in outcomes between treated spouses and their matched controls. 4) Preventive care items indicate whether the respondent reports receiving the service since the last survey wave: blood test for cholesterol, flu shot, monthly breast self-exam (women only), mammogram (women only), Pap smear (women only), and prostate cancer check (men only). Gender-specific items are coded as missing for ineligible respondents. Higher values indicate greater use/engagement. *** *p<0.01,* ** *p<0.05,* * *p<0.10.*

.

Table S10 Average Pre-Treatment Effects with Three-Wave Leads (~6 Years)

	ATT
**Self-reported poor Health**	-0.00
	(-0.02, 0.01)
**Number of observations**	29,097
**Number of chronic conditions**	-0.03
	(-0.06, 0.01)
**Number of observations**	29,097
**CES-D scale (0-8)**	-0.01
	(-0.08, 0.07)
**Number of observations**	27,396
**Number of ADL limitations (5 items)**	-0.02
	(-0.05, 0.01)
**Number of observations**	29,096
**Number of IADL limitations (5 items)**	-0.02
	(-0.05, 0.01)
**Number of observations**	29,096

Sources/Notes. 1) Sample is from the Health and Retirement Study (HRS), 1992–2020; opposite-sex married/cohabiting couples; one respondent per household-wave. 2) Index spouses experience a health shock (first report of cancer, stroke, or heart disease) at the index wave. 3) Matching: 1:1 nearest-neighbor matching without replacement conducted in the pre-event wave; exact matching on index spouse age, sex, race/ethnicity, education; non-index spouse age and education; and survey wave. 4) ATT = average treatment effect on the treated; estimates represent the average difference in outcomes between treated spouses and their matched controls. 5) The 8-item CES-D measures depressive symptoms over the past two weeks; scores range from 0 (no symptoms) to 8 (more symptoms). 6) ADL (0–5): bathing, dressing, eating, getting in/out of bed, and walking across a room. IADL (0–5): using the telephone, managing money, taking medications, grocery shopping, and preparing hot meals. Higher scores indicate more limitations. *** $p<0.01$, ** $p<0.05$, * $p<0.10$.

Table S11 Average Pre- and Post-Treatment Effects on Selection-Related Outcomes

	ATT
**Working for pay (binary)**	0.02*
	(0.00, 0.04)
**Average pre-treatment effects**	0.01
	(-0.01, 0.03)
**Number of observations**	28,988
**Work hours**	-0.62
	(-1.74, 0.50)
**Average pre-treatment effects**	-0.01
	(-1.06, 1.04)
**Number of observations**	16,001
**Hospital stay (binary)**	0.00
	(-0.02, 0.03)
**Average pre-treatment effects**	-0.02
	(-0.04, 0.01)
**Number of observations**	29,001
**Number of hospital nights**	0.06
	(-0.42, 0.54)
**Average pre-treatment effects**	-0.15
	(-0.69, 0.40)
**Number of observations**	28,981
**Out-of-pocket expenditures (USD)**	106.84
	(-306.44, 520.11)
**Average pre-treatment effects**	54.173
	(-331.62, 439.96)
**Number of observations**	29,004

Sources/Notes. 1) Sample is from the Health and Retirement Study (HRS), 1992–2020; opposite-sex married/cohabiting couples; one respondent per household-wave. 2) Index spouses experience a health shock (first report of cancer, stroke, or heart disease) at the index wave. 3) Matching: 1:1 nearest-neighbor matching without replacement conducted in the pre-event wave; exact matching on index spouse age, sex, race/ethnicity, education; non-index spouse age and education; and survey wave. 4) ATT = average treatment effect on the treated; estimates represent the average difference in outcomes between treated spouses and their matched controls. *** *p<0.01,* ** *p<0.05,* * *p<0.10.*

Table S12 Average Post-Treatment Changes in Spouses' Health Outcomes After Partner's Health Shock: Estimates by Individual Diagnosis

**All**	**Cancer, stroke, heart disease**	**Cancer only**	**Stroke only**	**Heart Disease only**
**Self-reported poor health**	0.02*	0.02*	0.03*	0.00
	(-0.00, 0.03)	(-0.00, 0.05)	(-0.01, 0.06)	(-0.02, 0.02)
**Number of observations**	29,165	8,458	5,250	15,457
**Number of chronic conditions**	0.05***	0.02	0.09**	0.05**
	(0.01, 0.08)	(-0.03, 0.07)	(0.02, 0.17)	(0.01, 0.09)
**Number of observations**	29,165	8,458	5,250	15,457
**High blood pressure**	0.00	-0.02**	0.02	-0.01
	(-0.01, 0.01)	(-0.04, 0.00)	(-0.01, 0.05)	(-0.02, 0.01)
**Number of observations**	29,165	8,458	5,250	15,457
**Diabetes**	0.00	-0.01	0.01*	0.01
	(-0.01, 0.02)	(-0.03, 0.01)	(-0.00, 0.03)	(-0.01, 0.03)
**Number of observations**	29,165	8,458	5,250	15,457
**Cancer**	0.01**	0.00	0.01	0.01
	(-0.00, 0.02)	(-0.01, 0.02)	(-0.02, 0.03)	(-0.01, 0.02)
**Number of observations**	29,165	8,458	5,250	15,457
**Lung diseases**	0.01	0.00	0.03***	0.01
	(-0.00, 0.02)	(-0.01, 0.01)	(0.01, 0.05)	(-0.01, 0.02)
**Number of observations**	29,165	8,458	5,250	15,457
**Heart diseases**	0.01	0.02**	-0.01	0.02**
	(-0.00, 0.02)	(0.00, 0.04)	(-0.03, 0.01)	(0.00, 0.03)
**Number of observations**	29,165	8,458	5,250	15,457
**CES-D scale (0-8)**	0.08**	0.08	0.14*	0.06
	(0.01, 0.16)	(-0.03, 0.19)	(-0.01, 0.29)	(-0.03, 0.14)
**Number of observations**	27,439	7,957	4,939	14,543
**Number of ADL limitations**	0.05**	0.04*	0.07*	0.05
	(0.01, 0.08)	(-0.00, 0.09)	(-0.01, 0.15)	(-0.00, 0.10)
**Number of observations**	29,163	8,457	5,249	15,456
**Number of IADL limitations**	0.04**	0.06**	0.04*	0.02
	(0.00, 0.07)	(0.01, 0.12)	(0.00, 0.09)	(-0.06, 0.10)
**Number of observations**	29,163	8,457	5,249	15,456

Sources/Notes. 1) Sample is from the Health and Retirement Study (HRS), 1992–2020; opposite-sex married/cohabiting couples; one respondent per household-wave. 2) Index spouses experience a health shock (first report of cancer, stroke, or heart disease) at the index wave. 3) Matching: 1:1 nearest-neighbor matching without replacement conducted in the pre-event wave; exact matching on index spouse age, sex, race/ethnicity, education; non-index spouse age and education; and survey wave. 4) The 8-item CES-D measures depressive symptoms over the past two weeks; scores range from 0 (no symptoms) to 8 (more symptoms). 5) ADL (0–5): bathing, dressing, eating, getting in/out of bed, and walking across a room. IADL (0–5): using the telephone, managing money, taking medications, grocery shopping, and preparing hot meals. Higher scores indicate more limitations. *** *p<0.01,* ** *p<0.05,* * *p<0.10.*

Table S13 Average Post-Treatment Changes in Spouses' Health Outcomes After Partner's Health Shock: Averages over Varying Post-Shock Windows

**Follow-up periods**	**1 wave (≈2 years)**	**2 waves (≈ 4 years)**	**3 waves (≈ 6 years; main model)**
**Self-reported poor health**	0.01	0.01*	0.02*
	(-0.01, 0.026)	(-0.00, 0.03)	(-0.00, 0.03)
**Number of observations**	28,863	28,988	29,004
**Number of chronic conditions**	0.03**	0.04***	0.05***
	(0.01, 0.06)	(0.01, 0.07)	(0.01, 0.08)
**Number of observations**	28,863	28,988	29,004
**CES-D scale (0-8)**	0.08**	0.07**	0.08**
	(0.00, 0.15)	(0.00, 0.15)	(0.01, 0.16)
**Number of observations**	27,159	27,291	27,298
**Feeling depressed (binary)**	0.01	0.01	0.01
	(-0.01, 0.03)	(-0.01, 0.02)	(-0.01, 0.02)
**Number of observations**	27,145	27,277	27,312
**Number of ADL limitations (5 items)**	0.05***	0.04**	0.05**
	(0.01, 0.08)	(0.01, 0.08)	(0.01, 0.08)
**Number of observations**	28,861	28,988	29,002
**Number of IADL limitations (5 items)**	0.03**	0.03*	0.04**
	(0.00, 0.06)	(-0.00, 0.06)	(0.00, 0.07)
**Number of observations**	28,861	28,985	29,002

Sources/Notes. 1) Sample is from the Health and Retirement Study (HRS), 1992–2020; opposite-sex married/cohabiting couples; one respondent per household-wave. 2) Index spouses experience a health shock (first report of cancer, stroke, or heart disease) at the index wave. 3) Matching: 1:1 nearest-neighbor matching without replacement conducted in the pre-event wave; exact matching on index spouse age, sex, race/ethnicity, education; non-index spouse age and education; and survey wave. 4) The 8-item CES-D measures depressive symptoms over the past two weeks; scores range from 0 (no symptoms) to 8 (more symptoms). 5) ADL (0–5): bathing, dressing, eating, getting in/out of bed, and walking across a room. IADL (0–5): using the telephone, managing money, taking medications, grocery shopping, and preparing hot meals. Higher scores indicate more limitations. *** $p<0.01$, ** $p<0.05$, * $p<0.10$.

Table S14 Main Definition (First Cancer/Stroke/Heart Disease) vs. Broad Definition (Hospital Stay ≥7 Days)

	First Onset of Cancer, Stroke, Heart Disease (Main model)	Extended Hospitalization (7 days or longer)
**Self-reported poor health**	0.02*	0.07*
	(-0.00, 0.03)	(-0.01, 0.15)
**Number of observations**	29,165	6,774
**Number of chronic conditions**	0.05***	0.11
	(0.01, 0.08)	(-0.06, 0.29)
**Number of observations**	29,165	6,774
**CES-D scale**	0.08**	0.25
	(0.01, 0.16)	(-0.14, 0.65)
**Number of observations**	27,439	6,319
**Number of ADL limitations (5 items)**	0.05**	0.18*
	(0.01, 0.08)	(-0.00, 0.37)
**Number of observations**	29,163	6,774
**Number of IADL limitations (5 items)**	0.04**	0.17*
	(0.00, 0.07)	(-0.00, 0.35)
**Number of observations**	29,163	6,774

Sources/Notes. 1) Sample is from the Health and Retirement Study (HRS), 1992–2020; opposite-sex married/cohabiting couples; one respondent per household-wave. 2) Index spouses experience a health shock (first report of cancer, stroke, or heart disease) at the index wave. 3) Matching: 1:1 nearest-neighbor matching without replacement conducted in the pre-event wave; exact matching on index spouse age, sex, race/ethnicity, education; non-index spouse age and education; and survey wave. 4) The 8-item CES-D measures depressive symptoms over the past two weeks; scores range from 0 (no symptoms) to 8 (more symptoms). 5) ADL (0–5): bathing, dressing, eating, getting in/out of bed, and walking across a room. IADL (0–5): using the telephone, managing money, taking medications, grocery shopping, and preparing hot meals. Higher scores indicate more limitations. *** $p<0.01$, ** $p<0.05$, * $p<0.10$.

Table S15 Average Post-Treatment Changes in Spouses' Health Outcomes by Time Since Partner's Health Shock

	Main specification (biannual)	Health shocks less than 12 months	Health shocks 12+ months
**Self-reported poor health (binary)**	0.02*	0.03*	0.05
	(-0.00, 0.03)	(0.00, 0.05)	(-0.02, 0.08)
**Number of observations**	29,165	18,082	9,712
**Number of chronic conditions**	0.05***	0.06**	0.09*
	(0.01, 0.08)	(0.01, 0.10)	(0.01, 0.16)
**Number of observations**	29,165	18,082	9,712
**High blood pressure**	0.00	0.02	0.04
	(-0.01, 0.01)	(-0.01, 0.03)	(-0.03, 0.05)
**Number of observations**	29,165	18,082	9,712
**Diabetes**	0.00	0.02	0.04
	(-0.01, 0.02)	(-0.01, 0.03)	(-0.02, 0.06)
**Number of observations**	29,165	18,082	9,712
**Cancer**	0.01**	0.02	0.03*
	(-0.00, 0.02)	(-0.01, 0.03)	(0.00 0.05)
**Number of observations**	29,165	18,082	9,712
**Lung diseases**	0.01	-0.01	0.01
	(-0.00, 0.02)	(-0.02, 0.00)	(-0.02, 0.04)
**Number of observations**	29,165	18,082	9,712
**Heart diseases**	0.01	-0.01	0.01
	(-0.00, 0.02)	(-0.02, 0.01)	(-0.01, 0.04)
**Number of observations**	29,165	18,082	9,712
**CES-D scale (0-8)**	0.08**	0.04***	0.15***
	(0.01, 0.16)	(0.01, 0.07)	(0.04, 0.27)
**Number of observations**	27,439	17,012	9,137
**ADL limitations**	0.05**	0.05**	0.09*
	(0.01, 0.08)	(0.01, 0.09)	(0.01, 0.16)
**Number of observations**	29,163	18,081	9,711
**IADL limitations**	0.04**	0.04**	0.05**
	(0.00, 0.07)	(0.01, 0.07)	(0.01, 0.09)
**Number of observations**	29,163	18,081	9,711

Notes. 1) Sample is from the Health and Retirement Study (HRS), 1992–2020; opposite-sex married/cohabiting couples; one respondent per household-wave. 2) Index spouses experience a health shock (first report of cancer, stroke, or heart disease) at the index wave. 3) Matching: 1:1 nearest-neighbor matching without replacement conducted in the pre-event wave; exact matching on index spouse age, sex, race/ethnicity, education; non-index spouse age and education; and survey wave. 4) The 8-item CES-D measures depressive symptoms over the past two weeks; scores range from 0 (no symptoms) to 8 (more symptoms). 5) ADL (0–5): bathing, dressing, eating, getting in/out of bed, and walking across a room. IADL (0–5): using the telephone, managing money, taking medications, grocery shopping, and preparing hot meals. Higher scores indicate more limitations. *** *p<0.01,* ** *p<0.05,* * *p<0.10.*

Table S16 Spousal Health Outcomes After Partner's Health Shock: Corrective ATT Weights vs. Unweighted Estimates

	No weight (main model)	Weighted
**Self-reported poor health (binary)**	0.02*	0.02*
	(-0.00, 0.03)	(-0.00, 0.03)
**Number of observations**	29,165	29,165
**Number of chronic conditions**	0.05***	0.04***
	(0.01, 0.08)	(0.01, 0.07)
**Number of observations**	29,165	29,165
**CES-D scale**	0.08**	0.08**
	(0.01, 0.16)	(0.01, 0.15)
**Number of observations**	27,439	27,439
**Number of ADL limitations (5 items)**	0.05**	0.04**
	(0.01, 0.08)	(0.01, 0.08)
**Number of observations**	29,163	29,163
**Number of IADL limitations (5 items)**	0.04**	0.03*
	(0.00, 0.07)	(0.00, 0.07)
**Number of observations**	29,163	29,163

Sources/Notes. 1) Sample is from the Health and Retirement Study (HRS), 1992–2020; opposite-sex married/cohabiting couples; one respondent per household-wave. 2) Index spouses experience a health shock (first report of cancer, stroke, or heart disease) at the index wave. 3) Matching: 1:1 nearest-neighbor matching without replacement conducted in the pre-event wave; exact matching on index spouse age, sex, race/ethnicity, education; non-index spouse age and education; and survey wave. 4) The 8-item CES-D measures depressive symptoms over the past two weeks; scores range from 0 (no symptoms) to 8 (more symptoms). 5) ADL (0–5): bathing, dressing, eating, getting in/out of bed, and walking across a room. IADL (0–5): using the telephone, managing money, taking medications, grocery shopping, and preparing hot meals. Higher scores indicate more limitations. *** *p<0.01,* ** *p<0.05,* * *p<0.10.*

Appendix S1. Definitions and Measurement of Outcomes and Covariates

Domain	Variable	Definition / Measurement	Coding / Range
**Exposure**			
**Health shocks**	Partner's major health shock	Indicator that the index spouse newly reported a doctor diagnosis of cancer, stroke, or heart disease during follow-up. The shock date was assigned to the interview wave in which the diagnosis was first reported. Exposure was treated as absorbing thereafter.	Binary indicator by wave
**Primary Outcomes**			
**Self-rated health**	Poor self-rated health	Non-index spouse's self-rated health recoded to distinguish fair/poor from excellent/very good/good.	1 = fair/poor; 0 = excellent/very good/good
**Chronic disease burden**	Number of chronic conditions	Count of doctor-diagnosed conditions among the following: high blood pressure, diabetes, cancer, lung disease, heart disease, stroke, arthritis, and psychiatric problems.	Count; higher values indicate greater morbidity
**Specific conditions**	Hypertension, diabetes, cancer, lung disease, heart disease	Binary indicators for each physician-diagnosed condition.	1 = condition present; 0 = absent
**Depressive symptoms**	CES-D (8-item)	Depressive symptoms measured using the 8-item Center for Epidemiologic Studies Depression Scale. Items include feeling depressed, everything being an effort, restless sleep, loneliness, sadness, inability to get going, happiness, and enjoyment of life. The two positive-affect items were reverse-coded and summed so that higher values reflect more symptoms.	0–8; higher values indicate more depressive symptoms
**Physical functioning**	ADL limitations	Count of difficulties with five activities of daily living: bathing, dressing, eating, getting in/out of bed, and walking across a room.	0–5; higher values indicate more limitations
**Instrumental functioning**	IADL limitations	Count of difficulties with five instrumental activities of daily living: using the telephone, managing money, taking medications, grocery shopping, and preparing hot meals.	0–5; higher values indicate more limitations
**Secondary outcomes**			
**Smoking**	Current smoking	Indicator for whether the non-index spouse currently smokes.	1 = current smoker; 0 = former/never
**Alcohol use**	Current drinking	Indicator for whether the non-index spouse currently drinks alcohol.	1 = current drinker; 0 = former/never
**Preventive care**	Preventive care use	Binary indicators for receipt of preventive services in the prior two years: cholesterol blood test, flu shot, breast self-exam, mammogram, Pap smear, and prostate cancer screening, as applicable by sex. Because some preventive care items were asked in alternating waves, the most recent valid response from the adjacent administered wave was carried forward when needed.	1 = received service; 0 = did not receive service

Domain	Variable	Definition / Measurement	Coding / Range
**Main covariates**			
	Respondent and spouse age	Time-varying age measures for both spouses.	Years
	Household assets	Inflation-adjusted household assets, standardized within wave.	Continuous
	Urbanicity	Indicator for urban versus rural residence.	1 = rural; 0 = urban
	Work status	Employment status of the non-index spouse.	1 = working for pay; 0 = not working for pay
	Number of children	Total number of children.	Count
**Supplementary adjustment covariates**			
	Baseline health conditions	Broader pre-event adjustment set including BMI, chronic conditions, functional limitations, and self-rated health.	BMI (continuous); number of chronic conditions (count of eight doctor-diagnosed conditions); ADL limitations (0–5 count); IADL limitations (0–5 count); self-rated health (1 = fair/poor; 0 = excellent/very good/good)
	Environmental context	Urban locality and state of residence.	Urban locality (1 = rural; 0 = urban); state of residence (categorical)
	Socioeconomic conditions	Household poverty.	1 = household income in the last calendar year was below the U.S. Census Bureau poverty threshold for the corresponding family composition and year; 0 = otherwise
	Marital history	Marriage duration.	Years
	Health care access	Government-based and employment-based insurance coverage.	Government-based insurance coverage (1 = covered by any government health insurance program, including Medicare, Medicaid, VA/CHAMPUS, or other government coverage; 0 = otherwise); employment-based insurance coverage (1 = covered by employer-provided health insurance through the respondent's or spouse's current or former employer, union, or own business; 0 = otherwise)
	Retirement status	Indicator of retirement status.	1 = retired; 0 = otherwise
	Physical activity	Indicator for frequent physical activity	1 = participation in light and/or vigorous physical activity 3 or more times per week; 0 = otherwise
**Matching covariates**			
**Key socio-demographics**	Index and non-index spouse age, sex, race/ethnicity, education	Time-varying age measures for both spouses; sex/gender; race/ethnicity	Years; 1 = male, 2 = female; 1= White/Caucasian, 2 = Black/African American, 3 = Other; years of schooling
**Survey wave**	Survey wave	Exact matching on calendar survey wave to align treatment and control observations in time.	Wave indicator

Appendix S2. Models

Staggered Event Study Design

We estimated the effect of a partner's incident diagnosis of cancer, stroke, or heart disease on the other spouse's subsequent health using a stacked event study design, which is well suited to tracing dynamic changes before and after the diagnosis while reducing bias from variation in event timing. For each qualifying diagnosis occurring at survey wave r, we constructed a seven-wave sub-panel covering $r - 3, \ldots, r + 3$ (e.g., a couple with a shock at wave 4 contributes a window spanning waves 1-7; a couple with a shock at wave 12 contributes a window spanning waves 9-15), yielding a balanced seven-wave panel centered on the event. Each window defines a separate "sub-experiment" in which treated couples are those whose shock occurs at r, and controls are couples whose shock occurs outside the window or never occurs during follow-up.

Our main specification is an event-study regression in the stacked sample:

$$Y_{it} = \sum_{r=-3, r\neq -1}^{+3} \beta_r 1\{k_{it} = r\} + \gamma^{\top} X_{i,t-1} + \alpha_i + \tau_t + \epsilon_{it}$$

where Y_{it} is the spouse health outcome for individual i at wave t, k_{it} is event time relative to the partner's shock (with $r = -1$ as the omitted category), $X_{i,t-1}$ are time-varying covariates measured one wave prior to the event window, α_i are individual fixed effects, and τ_t are time fixed effects (in waves). The main coefficients β_r trace dynamic effects before and after the shock and identify the average treatment effect on the treated (ATT) within the window. We assess parallel trends by testing whether average pre-treatment effects ($r = -3, -2;\ 4\ years$) equal zero and by visually inspecting event-time plots.

We then stack all sub-experiments into a single dataset and estimate event-time effects by comparing changes in outcomes for exposed spouses to changes for controls within the same

window, including individual fixed effects and time fixed effects to absorb time-invariant heterogeneity and common shocks. We defined treatment at the earliest qualifying diagnosis to prevent couples from re-entering control risk sets and to avoid contamination from anticipation (e.g., pre-diagnosis changes in the spouse's health or behavior) or subsequent overlapping diagnoses; when multiple conditions occur, we prioritize the first chronologically. This construction avoids the "forbidden comparisons" that bias two-way fixed-effects (TWFE) under staggered timing because treated units are compared only with never-treated (or not-yet-treated) units within the same event window, rather than implicitly using already-treated units as controls for later-treated units (i.e., comparing groups at different exposure durations).

Matching

To account for observable differences between treated and control groups, we implement 1:1 nearest-neighbor matching without replacement at the survey wave immediately prior to shock onset ($r - 1$) and then carry the matched pairs into the stacked sample. Index spouses are matched to control spouses observed in the same pre-event wave. We enforce exact matches on baseline characteristics: index spouse age, sex, race/ethnicity, education; non-index spouse age and education; and survey wave. The distance metric includes the index spouse's age, sex, race/ethnicity, education, and work status, plus the non-index spouse's age, education, race/ethnicity. This procedure targets the Average Treatment Effect on the Treated (ATT) and yields a comparison group composed of spouses from similarly composed couples who had not experienced a partner shock by the index wave. The resulting estimates can be interpreted as causal effects of partner health shocks under the assumptions that, within event windows, the timing of shocks is as good as random conditional on matching and controls, and that treated and control units would have followed parallel trends absent the shock.

Appendix S3. Pre-matching Statistics

Descriptive Statistics of Index Spouses Aged 51+ Years Before Matching, Health and Retirement Study, 1992-2020.

	Before Matching (PN-Wave Observations)			
	**Control Group**	**Treatment Group**	**Total**	**Test**
N (%)	33,898 (43%)	44,175 (57%)	78,073 (100%)	
Age, mean (sd)	66.32 (10.52)	68.91 (10.33)	67.78 (10.49)	<0.001
Sex/gender (%)	19,889 (59%)	20,989 (48%)	40,878 (52%)	<0.001
Race/ethnicity (%)				
1.White/Caucasian	27,119 (80%)	38,538 (87%)	65,657 (84%)	<0.001
2.Black/African American	4,501 (13%)	4,494 (10%)	8,995 (12%)	
3.Other	2,278 (7%)	1,143 (3%)	3,421 (4%)	
Years of education, mean (sd)	12.49 (3.34)	12.54 (3.12)	12.52 (3.22)	<0.001
Rural residence (vs. urban) (%)	10,309 (30%)	14,108 (32%)	24,417 (31%)	<0.001
Working for pay (%)	11,764 (35%)	11,656 (26%)	23,420 (30%)	<0.001
Number of children, mean (sd)	2.75 (1.83)	2.91 (1.81)	2.84 (1.82)	<0.001

Sources/Notes. 1) Sample is from the Health and Retirement Study (HRS), 1992–2020; opposite-sex married/cohabiting couples; one respondent per household–wave. 2) "Treatment" = index spouses experience a health shock (first report of cancer, stroke, or heart disease) at the index wave; "Control" = index spouses who had not experienced a qualifying shock by that wave (never or not yet). 3) p-values come from two-sample tests (t-tests for means; χ^2 for proportions).

Evaluation of Parallel Trends: Average Pre-Treatment Effects Before Matching

	All	Women (index spouse: men)	Men (index spouse: women)
**Self-reported poor health (binary)**	-0.00	-0.02	0.02
	(-0.02, 0.01)	(-0.04, 0.00)	(-0.01, 0.04)
**Number of observations**	294,082	147,306	146,776
**Number of observations**	40,147	18,765	21,648
**Number of chronic conditions**	-0.02	-0.03	-0.03
	(-0.05, 0.01)	(-0.06, 0.01)	(-0.07, 0.02)
**Number of observations**	294,286	147,382	146,904
**Number of observations**	40,161	18,779	21,648
**CES-D scale**	-0.01	-0.06	0.06
	(-0.09, 0.06)	(-0.16, 0.04)	(-0.05, 0.17)
**Number of observations**	267,093	140,899	126,194
**Number of observations**	37,443	18,364	19,303
**Number of ADL limitations**	-0.02	0.00	-0.07**
	(-0.05, 0.01)	(-0.04, 0.04)	(-0.12, -0.01)
**Number of observations**	294,211	147,351	146,860
**Number of observations**	40,159	18,778	21,647
**Number of IADL limitations**	-0.02	-0.00	-0.06*
	(-0.05, 0.01)	(-0.03, 0.03)	(-0.11, 0.00)
**Number of observations**	294,210	147,351	146,859
**Number of observations**	40,159	18,778	21,647

Sources/Notes. 1) Sample is from the Health and Retirement Study (HRS), 1992–2020; opposite-sex married/cohabiting couples; one respondent per household–wave. 2) Index spouses experience a health shock (first report of cancer, stroke, or heart disease) at the index wave. 3) The reported coefficient for each outcome is the Average Pre-Treatment Effect, computed as the mean of all pre-event lead coefficients from the stacked event-study specification, *** *p<0.01,* ** *p<0.05,* * *p<0.10.*

Appendix S4. Sensitivity Analyses for Sample Restrictions

Because several sample restrictions could affect generalizability or introduce selection into the analytic sample, we conducted a series of sensitivity analyses to assess the robustness of the main findings. Specifically, we re-estimated the models under alternative specifications that: 1) included dyads in which both partners experienced qualifying health shocks; 2) included same-sex couples; 3) used a less restrictive unbalanced event-study design that allowed skip-and-return patterns; and 4) censored respondents at partnership dissolution rather than continuing follow-up after separation, divorce, or widowhood.

Across alternative sample specifications, the main pattern of results remained substantively similar. Including dyads in which both partners experienced qualifying health shocks did not materially change the overall conclusions, although effect sizes were somewhat larger for several physical health outcomes, including self-reported poor health, number of chronic conditions, cancer, and heart disease, while associations for depressive symptoms and IADL limitations were attenuated and less precise. Including same-sex couples had little effect on the estimates, which remained nearly identical in direction, magnitude, and statistical significance to the main specification. Likewise, relaxing the balanced-panel requirement to allow skip-and-return patterns did not materially alter the results, although estimates were modestly larger for some outcomes, particularly self-reported poor health, number of chronic conditions, and CES-D symptoms. Finally, censoring respondents at partnership dissolution produced estimates similar in direction and magnitude to the main specification, with slight attenuation for some outcomes. These results suggests that our analyses are primarily driven by the vast majority of the analytic sample that remains married or partnered across the study period.

Sensitivity Analyses of Post-Treatment Changes in Spouses' Health Outcomes Under Alternative Sample Restrictions

	Main specification	Including dual health shocks households	Including same-sex couples	Including respondents with skip-and-return patterns	Excluding respondents in partnership dissolutions (≈8%)
**Self-reported poor health**	0.02*	0.05**	0.02*	0.03*	0.02**
	(-0.00, 0.03)	(0.00, 0.10)	(0.00, 0.03)	(0.01, 0.04)	(0.00 0.03)
**Number of observations**	29,165	37,040	29,282	40,159	26,841
**Number of Chronic Conditions**	0.05***	0.10***	0.05***	0.07***	0.04***
	(0.01, 0.08)	(0.04 0.15)	(0.02, 0.08)	(0.03, 0.11)	(0.01, 0.07)
**Number of observations**	29,165	37,040	29,282	40,159	26,841
**High blood pressure**	0.00	0.00	0.00	0.00	0.00
	(-0.01, 0.01)	(-0.01, 0.01)	(-0.01, 0.01)	(-0.01, 0.01)	(-0.01, 0.01)
**Number of observations**	29,165	37,040	29,282	40,159	26,841
**Diabetes**	0.00	0.000	0.00	0.01	0.00
	(-0.01, 0.02)	(-0.01, 0.01)	(0.00, 0.02)	(0.00, 0.019)	(-0.01, 0.01)
**Number of observations**	29,165	37,040	29,282	40,159	26,841
**Cancer**	0.01**	0.02***	0.01**	0.01**	0.01**
	(-0.00, 0.02)	(0.01, 0.02)	(0.00, 0.02)	(0.00, 0.02)	(0.00, 0.02)
**Number of observations**	29,165	37,040	29,282	40,159	26,841
**Lung diseases**	0.01	0.00	0.01***	0.01*	0.01
	(-0.00, 0.02)	(-0.01, 0.00)	(-0.00, 0.02)	(0.00, 0.02)	(-0.00, 0.02)
**Number of observations**	29,165	37,040	29,282	40,159	26,841
**Heart diseases**	0.01	0.02***	0.01	0.01	0.01
	(-0.00, 0.02)	(0.02, 0.03)	(-0.00, 0.02)	(-0.00, 0.02)	(-0.01, 0.02)
**Number of observations**	29,165	37,040	29,282	40,159	26,841
**CES-D scale (0-8)**	0.08**	0.01	0.07**	0.10***	0.06**
	(0.01, 0.16)	(-0.11, 0.14)	(0.01, 0.13)	(0.04, 0.17)	(0.01, 0.12)
**Number of observations**	27,439	37,040	29,282	38,937	26,841
**ADL limitations**	0.05**	0.03**	0.05***	0.04***	0.04***
	(0.01, 0.08)	(0.00, 0.06)	(0.02, 0.07)	(0.01, 0.07)	(0.02, 0.07)
**Number of observations**	29,163	37,040	29,280	40,138	26,532
**IADL limitations**	0.04**	0.03	0.03**	0.03*	0.03**
	(0.00, 0.07)	(-0.03, 0.10)	(0.01, 0.06)	(0.00, 0.06)	(0.01, 0.06)
**Number of observations**	29,163	37,040	29,280	40,138	26,532

Notes. 1) Sample is drawn from the Health and Retirement Study (HRS), 1992–2020. The main specification is restricted to opposite-sex married or cohabiting couples and includes one respondent per household-wave; alternative columns modify these restrictions as indicated. 2) Index spouses experience a qualifying health shock, defined as the first report of cancer, stroke, or heart disease, at the index wave. 3) Matching uses 1:1 nearest-neighbor matching without replacement in the pre-event wave, with exact matching on index spouse age, sex, race/ethnicity, and education; non-index spouse age and education; and survey

wave. 4) CES-D is the 8-item depressive symptom scale (range: 0–8), with higher scores indicating more symptoms. 5) ADL and IADL limitations each range from 0 to 5; higher scores indicate more limitations. 6) Alternative specifications respectively: include households in which both partners experienced qualifying health shocks, include same-sex couples, allow respondents with skip-and-return patterns in an unbalanced event-study specification, or censor respondents at partnership dissolution rather than continuing follow-up after separation, divorce, or widowhood. *** $p<0.01$, ** $p<0.05$, * $p<0.10$.

Appendix S5. Robustness Tests

Pretrends. We first estimated event-time models with multiple leads (two waves, ≈4 years) , graphed the coefficients, and computed the average pre-treatment effect. As shown in **Table S2** and **Figure 1**, the lead estimates cluster tightly around zero and the average pre-treatment effects are statistically indistinguishable from zero, indicating no detectable anticipation or differential pretrends. We also repeated the lead/lag exercise 1) using longer leads of three waves (≈6 years) and 2) for variables that could signal selection, including working for pay, hours worked, any hospitalization, number of hospital nights, and out-of-pocket medical spending in the last 12 months. Results show no meaningful pre-treatment drift: average pre-treatment effects are statistically insignificant with longer leads and across all measures (**Table S10**, **Table S11**).

Sensitivity to post-shock windows. We re-estimated average treatment effects over alternative post-shock horizons, pooling outcomes across one, two, and three waves to assess sensitivity to window length and potential compositional change (**Table S13**). Results are remarkably stable across 1-, 2-, and 3-wave follow-ups. Spouses are more likely to report 'poor health; the decline is marginal in the first post-shock wave and reaches borderline significance by the second and third waves. Diagnosed morbidity rises persistently – the count of chronic conditions increases and the magnitude grows with longer follow-up, consistent with accumulating detection/management after the partner's diagnosis. Depressive symptoms (CES-D) increase modestly but consistently across windows. Functional limitations also worsen, particularly for ADLs; IADL effects are smaller and less precise.

Sensitivity to treatment definition. We assessed treatment-definition sensitivity by contrasting our acute definition (first stroke, incident cancer, heart disease) with a broader definition (extended length of stay in hospitals; 7 days or longer) (**Table S14).** Under the broader

definition, effects point in the same direction but are generally larger – for example, bigger declines in self-rated health and larger ADL/IADL estimates – while precision declines and most estimates are not statistically distinguishable from zero. This pattern likely reflects the hospitalization definition capturing more severe episodes (≥7 days, above the 90th percentile or higher) and applying to a much smaller sample, which yields wider intervals. Overall, the results suggest that spousal health consequences are not transitory and may be substantially larger for severe index events requiring longer hospitalizations.

Corrective Weights. Recent literature shows that basic stacked models (i.e., stacking sub-experiments and running a single pooled regression in the difference-in-differences/event-study framework) implicitly weight treated and control trends differently across stacks, yielding estimates that need not coincide with the average treatment effect on the treated (ATT). Wing et al. (2024) shows the bias can be removed by applying corrective sample weights so that the composition of treated and control observations is balanced across event times; the weighted stacked regression then identifies a trimmed aggregate ATT. Following this, we conduct a robustness test by implementing corrective sample weights that set treated observations to weight one and reweight controls by the ratio $(N_a^D/N^D)/(N_a^C/N^C)$, where N_a^D and N_a^C are, respectively, the numbers of treated and control units contributing to sub-experiment a, and N^D and N^C are the corresponding totals across all sub-experiments. This adjustment aligns the effective composition of control observations with that of the treated across stacks and restores identification of the ATT (**Table S16)**.

Supplementary Data Reference

1. Wing C, Freedman SM, Hollingsworth A. Stacked difference-in-differences. NBER Working Paper No. 32054. Cambridge, MA: National Bureau of Economic Research; 2024. doi:10.3386/w32054.
